# Diversity and paleoenvironmental implications of an elasmobranch assemblage from the Oligocene–Miocene boundary of Ecuador

**DOI:** 10.7717/peerj.9051

**Published:** 2020-04-29

**Authors:** Jorge D. Carrillo-Briceño, Jaime A. Villafaña, Carlos De Gracia, F. Fernando Flores-Alcívar, René Kindlimann, Juan Abella

**Affiliations:** 1 Palaeontological Institute and Museum, University of Zurich, Zurich, Switzerland; 2 University of Vienna, Department of Paleontology, Vienna, Austria; 3 Centro de Investigación en Recursos Naturales y Sustentabilidad, Universidad Bernardo O’Higgins, Santiago, Chile; 4 Center of Tropical Paleoecology and Archaeology, Smithsonian Tropical Research Institute, Panama, Panama; 5 Universidad Estatal de la Peninsula de Santa Elena, La Libertad, Santa Elena, Ecuador; 6 Institut Català de Paleontologia Miquel Crusafont, Universitat Autònoma de Barcelona, Cerdanyola del Vallès, Barcelona, Spain; 7 Instituto Nacional de Biodiversidad, (Parque La Carolina) Quito, Ecuador

**Keywords:** Eastern Central Pacific, Neogene, Sharks, Rays, Paleoenvironments, Tropical America, Fossils

## Abstract

The occurrence and diversity of elasmobranchs from the Oligocene–Miocene boundary from Tropical America is poorly known in comparison with the paleodiversity from younger Neogene intervals of the region. Here we describe a new elasmobranch assemblage from the rich fossil site of Montañita-Olón (Dos Bocas Formation, Santa Elena, Ecuador), where other vertebrates have already been described: for example, sea turtles and cetaceans. We report a total of 27 elasmobranch taxa, 19 of which are new fossil records for Ecuador, 10 new records for the Central Eastern Pacific and four new records for South America. Additionally, in order to reconstruct the environment where these marine remains were deposited, we performed abundance, paleobathymetric and habitat preference analyses, concluding that they were likely deposited in an outer neritic (open shelf) environment. The study of Oligocene and early Miocene marine elasmobranchs faunas in Tropical America is key to addressing the issues in the evolutionary history of this group.

## Introduction

**Figure 1:**
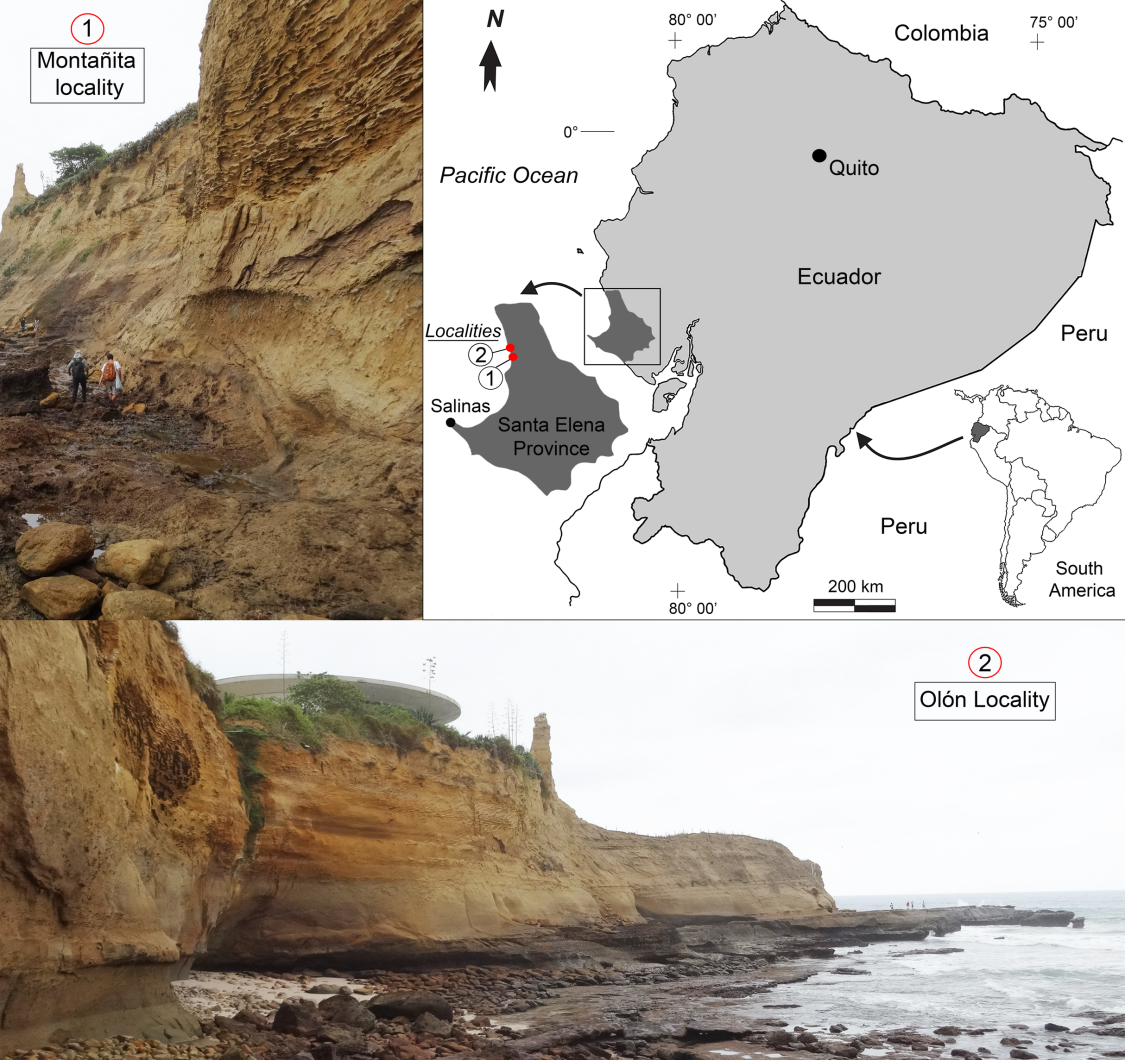
Location map of the fossiliferous localities of the Montañita-Olón outcrops (Dos Bocas Formation).

The Oligocene–Miocene transition (OMT) was an important period for the evolutionary history of the marine biota in the northern margins of South America, especially due to the significant changes that affected marine biota in the Pacific and proto-Caribbean region (Johnson, Sánchez-Villagra & Aguilera, 2009). At that time, large scale geological processes like the closure of the Central American Seaway (CAS) and the rise of the Panamanian Isthmus had not yet been completed (Coates & Stallard, 2013; Jaramillo et al., 2017). The CAS was a deep oceanic connection along the tectonic boundary between the Caribbean and South American plates that connected the Eastern Central Pacific (ECP) and Western Central Atlantic (WCA) (Jaramillo et al., 2017). This marine corridor allowed the flow of species (Leigh, O’Dea & Vermeij, 2014) until the end of the Miocene (Coates & Stallard, 2013; Bacon et al., 2015; Jaramillo et al., 2017). In this context, the evolutionary history of the marine fish fauna in Tropical America, according to the fossil record, has been linked with the geographic changes of the oceanic pathway that connected the ECP and WCA (e.g., Aguilera Socorro et al., 2011; Aguilera et al., 2017b; Schwarzhans & Aguilera, 2013; Carrillo-Briceño et al., 2018, and references therein). The study of Oligocene and early Miocene marine elasmobranch faunas is a proxy that allows us to address issues in evolutionary history in Tropical America, offering new light on factors that drove changes in biogeographic patterns of elasmobranchs before the closure of the CAS (Carrillo-Briceño et al., 2018). This oceanographic event resulted in a barrier that isolated the marine biota in both oceanic regions (ECP and WCA) (Lessios, 2008; Coates & Stallard, 2013; Leigh, O’Dea & Vermeij, 2014). Despite this, the Oligocene and early Miocene elasmobranch diversity from Tropical America is poorly known in comparison with the paleodiversity from younger Neogene intervals of the region (Carrillo-Briceño et al., 2018, fig. 5, tables S3 and S4). Oligocene elasmobranchs from Tropical America include a few isolated reports from the Caribbean region (Casier, 1958, 1966; Kruckow & Thies, 1990). In contrast, early Miocene assemblages from Tropical America are well known from the Caribbean and other WCA basins (Leriche, 1938; Casier, 1958; Santos & Travassos, 1960; Santos & Salgado, 1971; Iturralde-Vinent, Hubbell & Rojas, 1996; Sánchez-Villagra et al., 2000; Aguilera, 2010; Carrillo-Briceño et al., 2016a, 2016b, 2019; Aguilera et al., 2017a), as well as from the ECP in Peru (De la Cruz, 2008; Shimada et al., 2017; Landini et al., 2019). In this study, we present a revision of a new elasmobranch assemblage from the Oligocene–Miocene boundary (Dos Bocas Formation) of the Santa Elena Province, Ecuador, on the margin of the ECP (Fig. 1). The assemblage composition was studied, and we present a taxonomic list of sharks and rays with a comprehensive paleoenvironmental interpretation based on their bathymetric affinities. Additionally, the significance of the fossil assemblage for chronostratigraphic inferences is also discussed. The new elasmobranch assemblage, among other marine vertebrates found in the Dos Bocas Formation, including sea turtles (Cadena, Abella & Gregori, 2018), a new genus dolphin (Tanaka et al., 2017), and actinopterygian remains, increases the fossil record of the region and represents a critical window into marine tropical vertebrate faunas in the ECP during the OMT.

## Geological setting

**Figure 2:**
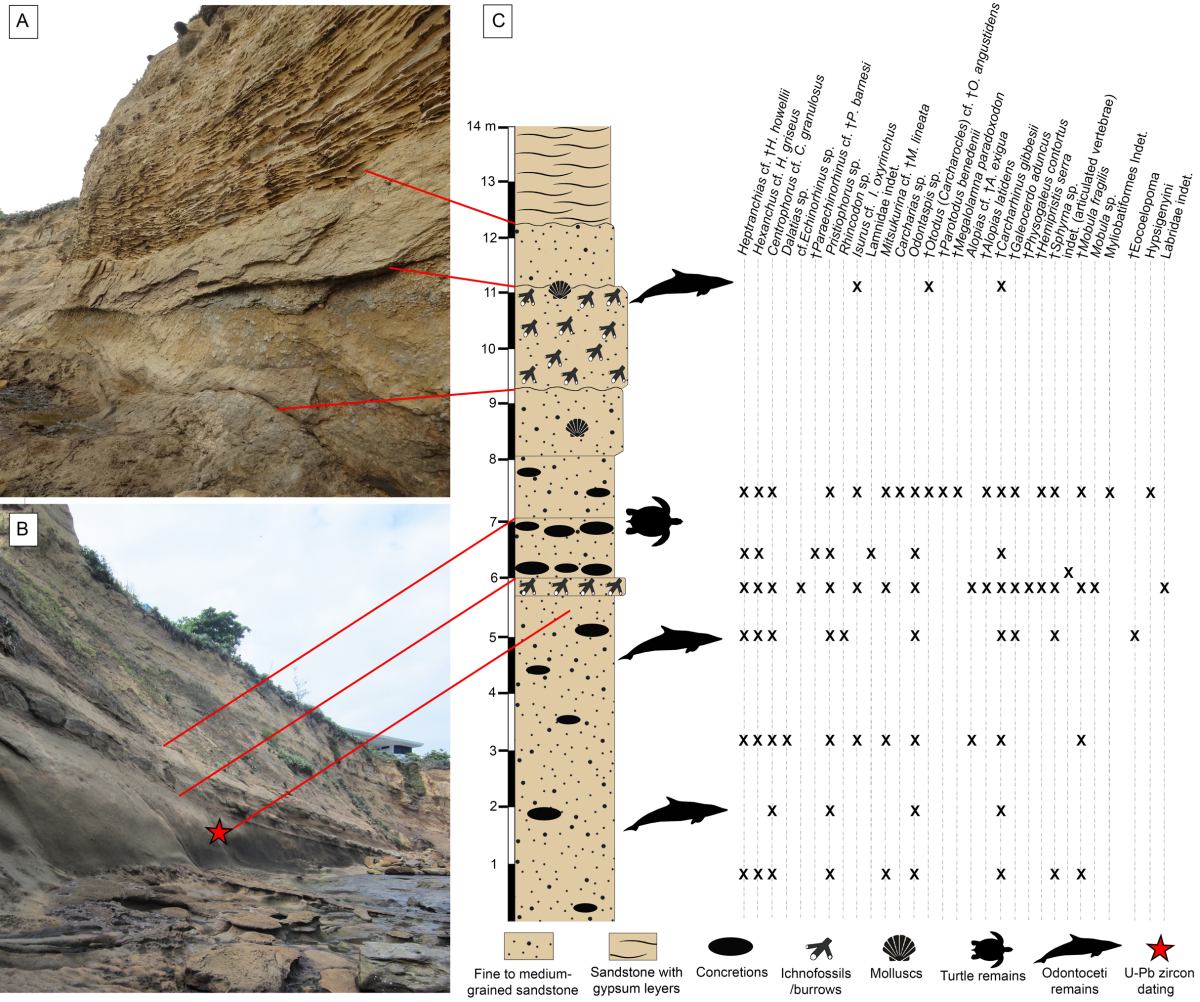
Stratigraphic context of the Montañita-Olón site. (A and B) Outcrops in the Montañita area. (C) Section showing stratigraphic provenance of fossil specimens. U-Pb zircon dating corresponds with the sample CP705 (age of 23.5 ± 0.4 Ma, with a younger cluster average of 22.9 ± 0.6 Ma) referred by Witt et al. (2019).

The outcrops of the Dos Bocas Formation studied herein correspond to a cliff of approximately 800 m in length along the coastline between the towns of Montañita and Olón (Fig. 1). Whittaker (1988) and Tanaka et al. (2017) provisionally identified the Montañita-Olón outcrops as part of the Zapotal Member of the Dos Bocas Formation. The fossil sharks and rays studied herein are from several points along the Montañita-Olón cliff, in localities that can only be accessed during low tides. For this study, a general stratigraphic column for the Montañita-Olón outcrops with an approximate thickness of 14 m was elaborated (Figs. 2A–2C). The geological section is dominated by strata composed of a moderately sorted fine to medium-grained sandstone with angular quartz-feldspathic clasts and rounded green grains (probably glauconite, although berthierine cannot be dismissed), with a micritic and volcanogenic matrix (Tanaka et al., 2017). Concretions are abundant in the section, even forming well-defined layers (Fig. 2B). In these concretions, other fossil vertebrates such as cetaceans, sea turtles and bony fishes have been collected (Tanaka et al., 2017; Cadena, Abella & Gregori, 2018). A well-defined thin bioturbated layer, with abundant fossil teeth is present underlying the above-mentioned concretion layer (Figs. 2B and 2C). Our observations confirm that bivalves and gastropods with poor preservation are also present, occurring in greater abundance in the upper layers of the section (Figs. 2A and 2C). It has been suggested as “general interpretation” that the Dos Bocas Formation was deposited in a shallow protected environment (see Tanaka et al., 2017; and references therein); although no clear evidence has been presented to justify this hypothesis for the Montañita-Olón outcrops. In contrast, an upper platform environment based on micropaleontological evidence was suggested for the Dos Bocas Formations by Ordoñez, Jiménez & Suárez (2006) and Witt et al. (2019, fig. 3C). A late Oligocene age has been inferred for the Dos Bocas Formation based on its faunal composition (Olsson, 1931; Bristow, 1975; Tanaka et al., 2017; Cadena, Abella & Gregori, 2018). A recent U-Pb zircon dating for the Montañita-Olón outcrops confirmed an age of 23.5 ± 0.4 Ma, with a younger cluster average of 22.9 ± 0.6 Ma (Witt et al., 2019). The sample used for the above mentioned U-Pb zircon dating, and referred by Witt et al. (2019) as “sample CP705”, was collected by one of the authors (JA) in the outcrops of Montañita area (Figs. 2B and 2C).

## Materials and Methods

**Figure 3:**
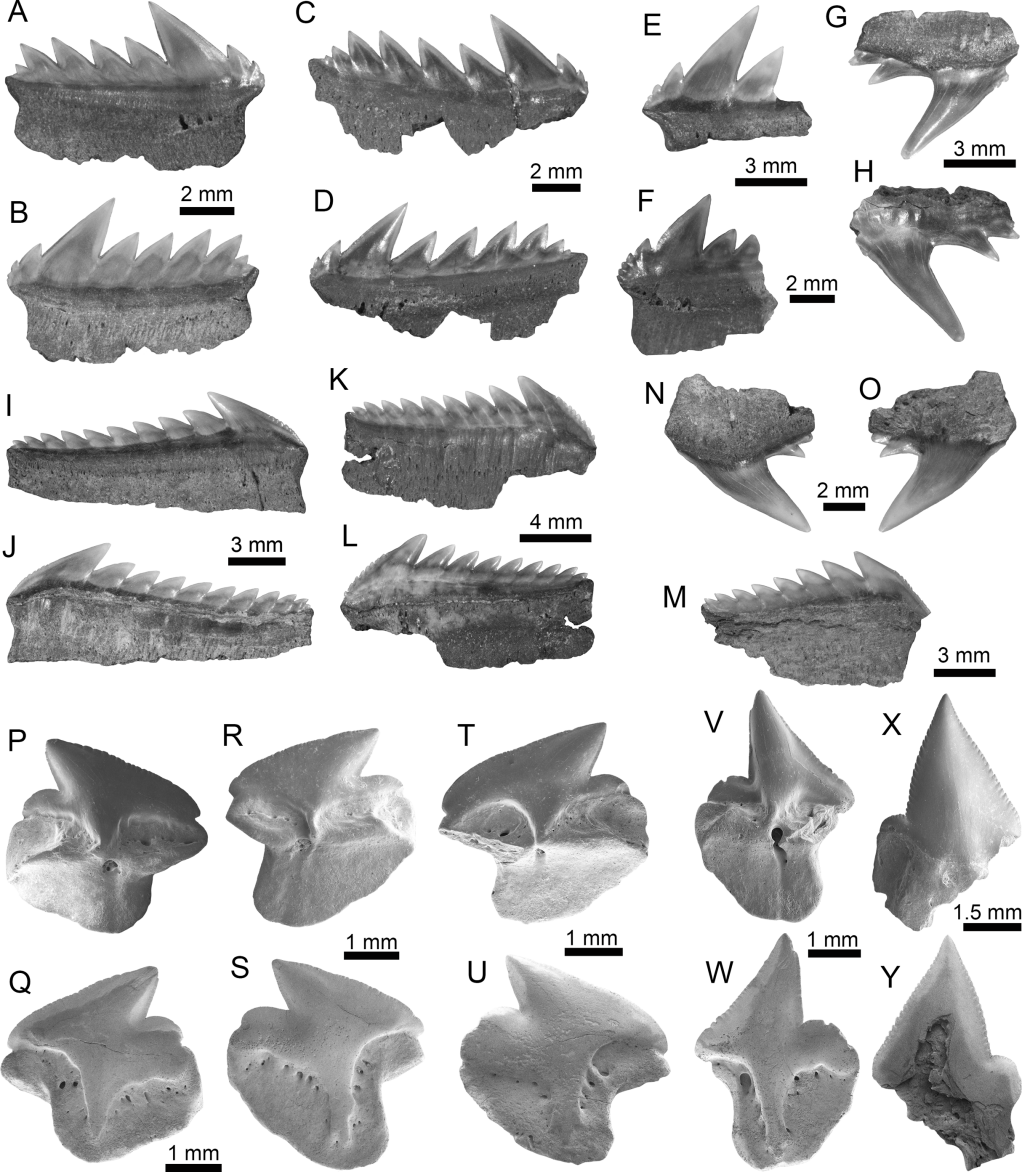
Hexanchiformes and Squaliformes of the Montañita-Olón site (Dos Bocas Formation). (A–H) *Heptranchias* cf. *H*. *howellii* ((A–H): MPM-1365; lower lateral teeth (A–F); upper lateral tooth (G and H)). (I–O) *Hexanchus* cf. *H. griseus* ((I–O): MPM-1359; lower lateral teeth (I–M); upper tooth (N and O)). (P–W) *Centrophorus* cf. *C*. *granulosus* ((P–W): MPM-1367; lower antero-lateral teeth (P–U); upper antero-lateral tooth (V–W)). (X and Y) *Dalatias* sp. (lower tooth (MPM-1366)). View: labial (B, C, H, J, K, O, Q, S, U, W and Y) and lingual (A, D–G, I, L–N, P, R, T, V and X).

**Figure 4:**
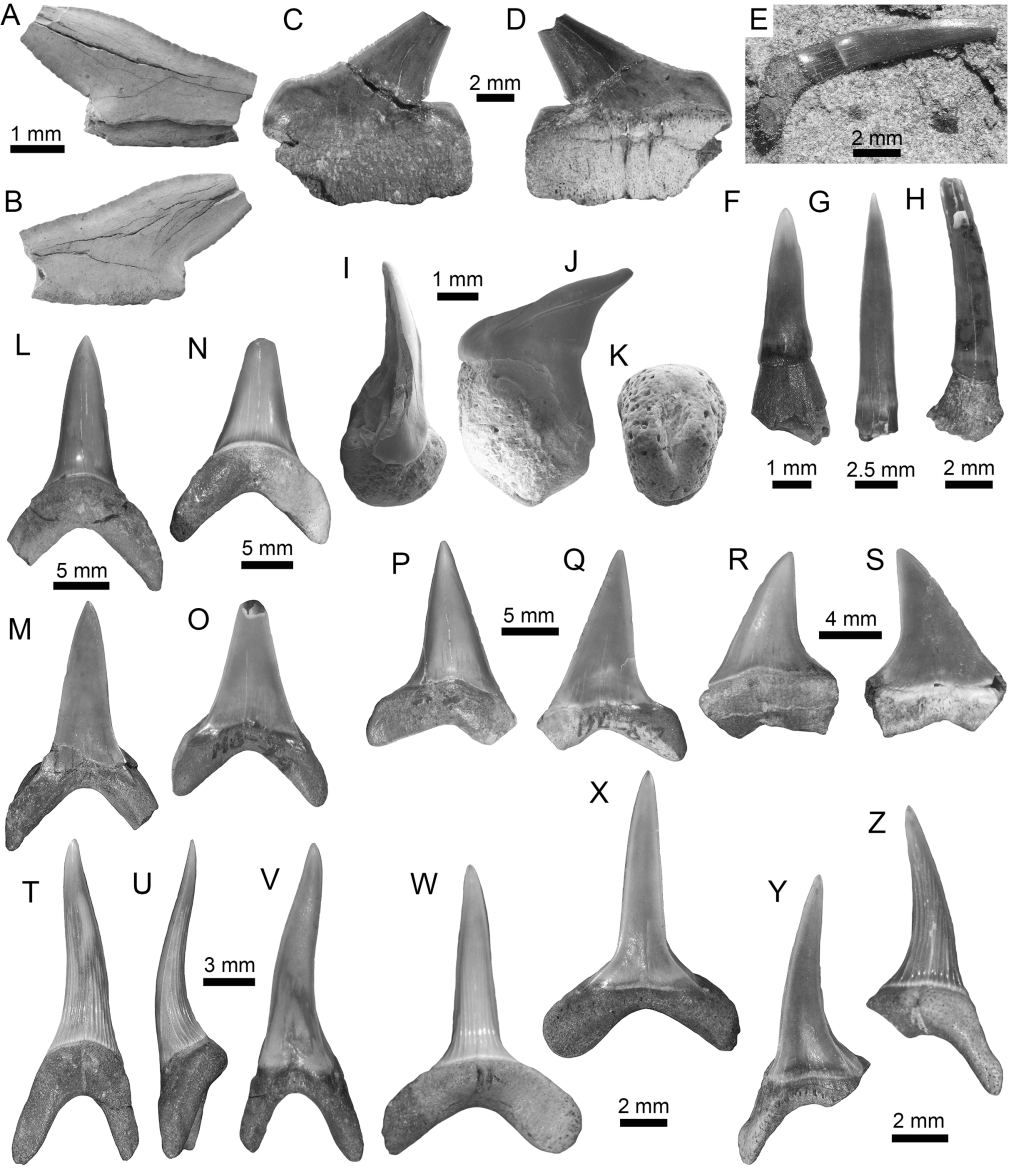
Echinorhiniformes, Pristiophoriformes, Orectolobiformes and Lamniformes of the Montañita-Olón site (Dos Bocas Formation). (A and B) cf. *Echinorhinus* sp. (antero-lateral tooth (MPM-1368)). (C and D) *Paraechinorhinus* cf. *P*. *barnesi* (antero-lateral tooth (MPM-1369)). (E–H) *Pristiophorus* sp. (rostral spines (MPM-1361)). (I–K) *Rhincodon* sp. (upper antero-lateral tooth (MPM-1370)). (L–S) *Isurus* cf. *I*. *oxyrinchus* ((L–S): MPM-1364; lower anterior teeth (L–O); lower tooth (P and Q); upper lateral tooth (R and S)). (T–Z) *Mitsukurina* cf. *M*. *lineata* ((T–Z): MPM-1371; lower anterior tooth (T–V); lower lateral tooth (W and X); upper anterior tooth (Y and Z)). View: labial (B, C, I, M, O, Q, S, V, X and Y), lingual (A, D, L, N, P, R, T, W and Z), dorsal (E–H) and profile (J and U), basal (k).

**Figure 5:**
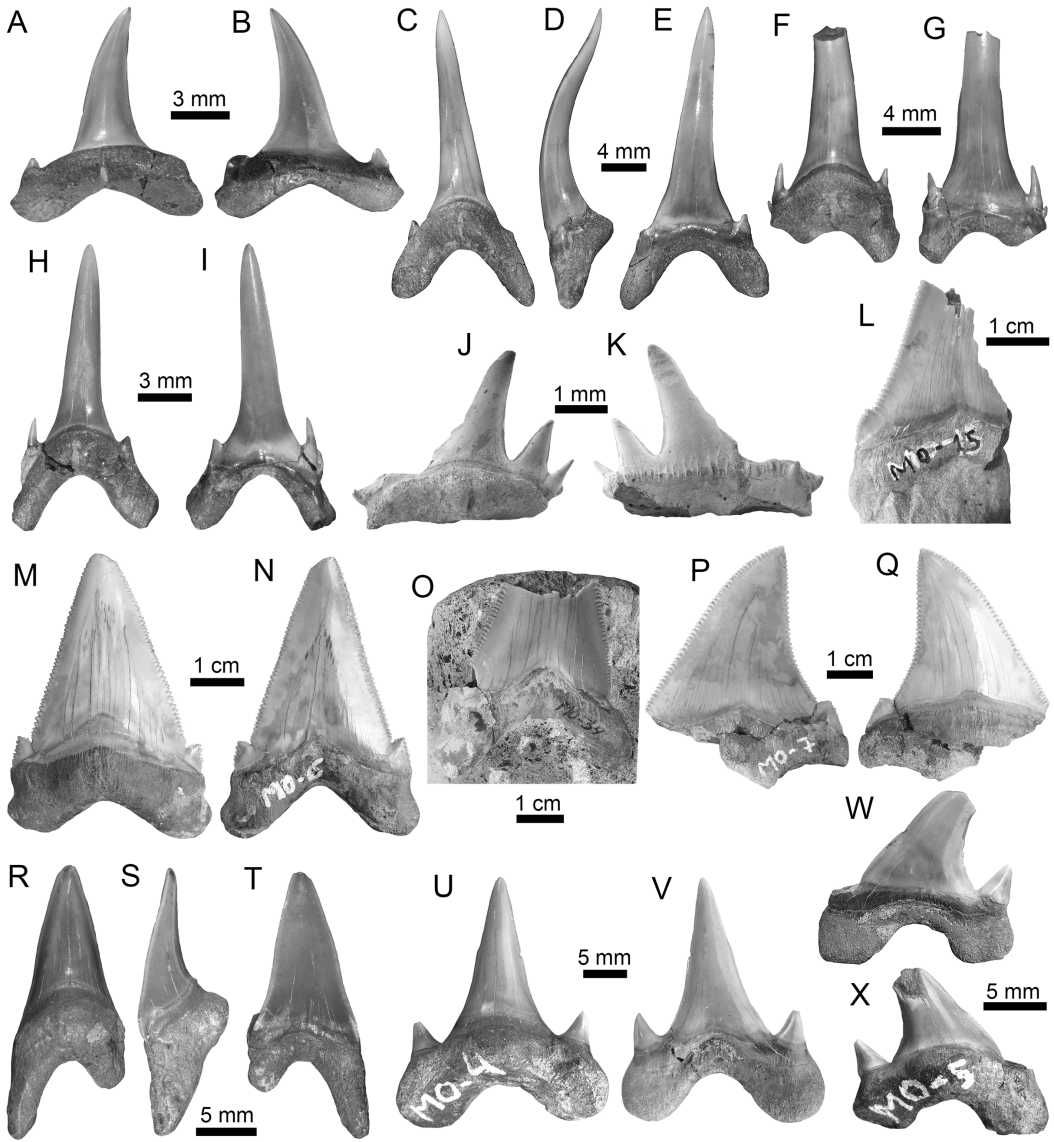
Lamniformes of the Montañita-Olón site (Dos Bocas Formation). (A and B) *Carcharias* sp. (Upper posterior tooth (MPM-1372)). (C–K) *Odontaspis* sp. ((C–K): MPM-1355; lower anterior teeth (C–I); upper posterior tooth (J and K)). (L–Q) *Otodus* (*Carcharocles*) cf. *O*. *angustidens* (L: lower anterior tooth (MPM-1356); (M and O): upper anterior teeth (MPM-1352; MPM-1360); (P and Q): upper lateral teeth (MPM-1353)). (R–T) *Parotodus benedenii* (presumed parasymphyseal tooth (MPM-1357)). (U–X) *Megalolamna paradoxodon* ((U and V): lower anterior tooth (MPM-1350); (W and X): upper lateral tooth (MPM-1351)). View: labial (B, E, G, I, K-L, N, O, P, T, V and W), lingual (A, C, F, H, J, M, Q, R, U and X) and profile (D and S).

**Figure 6:**
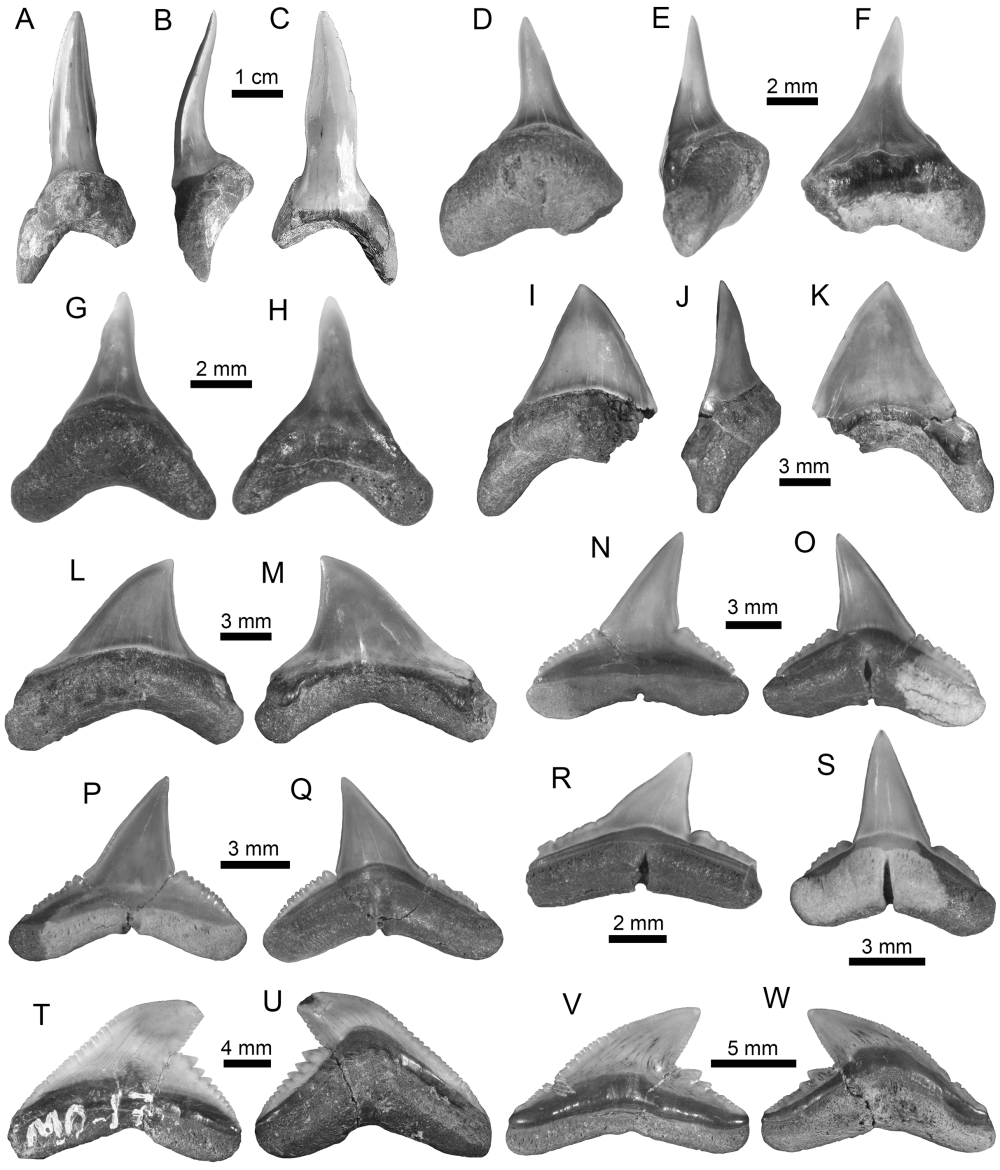
Lamniformes and Carcharhiniformes of the Montañita-Olón site (Dos Bocas Formation). (A–C) Lamnidae indet. (Lower anterior tooth (MPM-1377)). (D–H) *Alopias* cf. *A*. *exigua* (lower anterior teeth (MPM-1374)). (I–M) *Alopias latidens* (anterior lateral teeth (MPM-1375)). (N–S) *Carcharhinus gibbesii* (upper antero-lateral teeth (MPM-1376)). (T–W) *Galeocerdo aduncus* (antero-lateral teeth (MPM-1379)). View: labial (C, F, H, K, M, N, P, T and V), lingual (A, D, G, I, L, O, Q–S, U and W) and profile (B, E and J).

**Figure 7:**
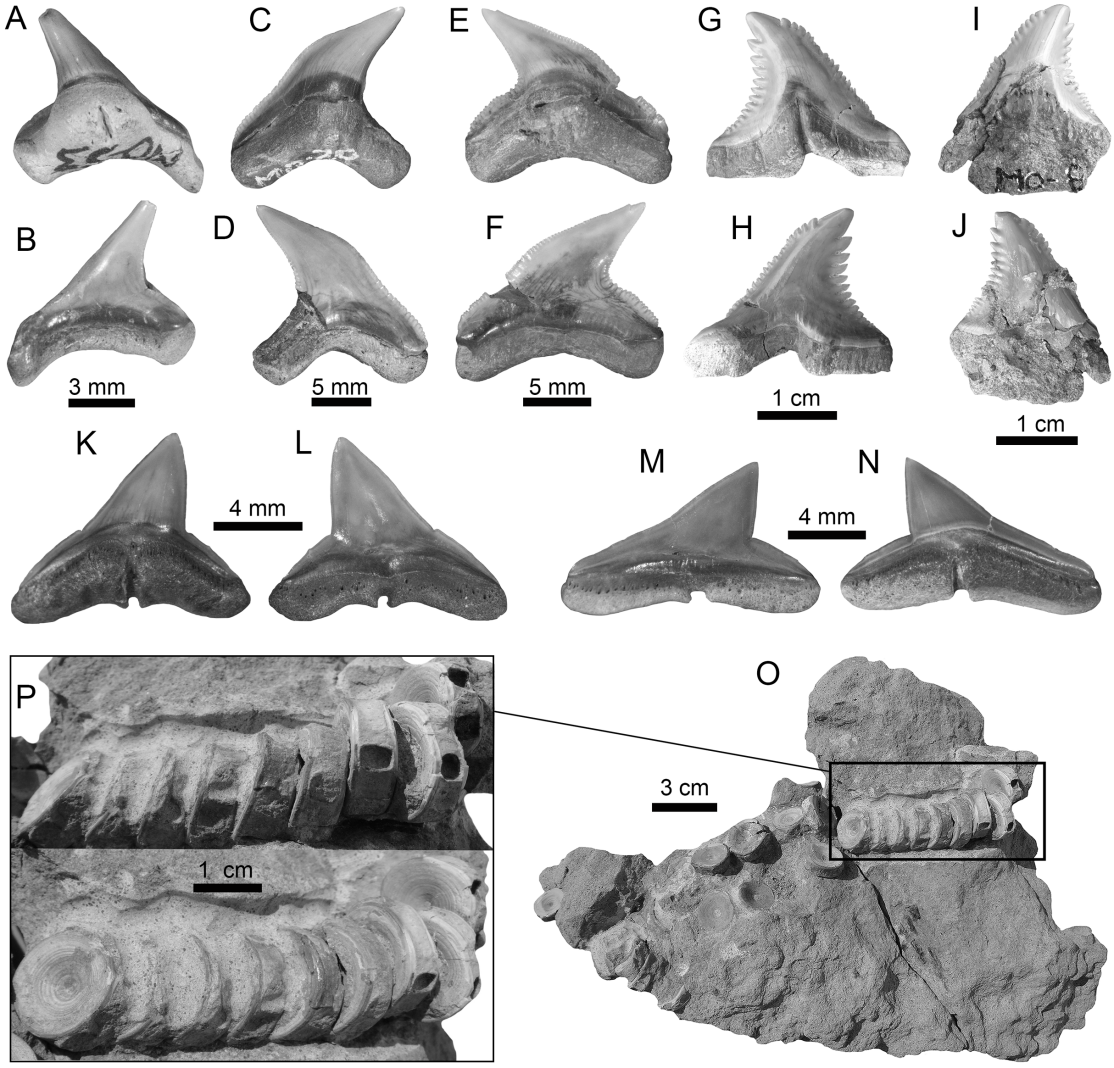
Carcharhiniformes of the Montañita-Olón site (Dos Bocas Formation). (A–F) *Physogaleus contortus* ((A–F): MPM-1380; lower antero-lateral tooth (A–D); upper antero-lateral tooth (E and F)). (G–J) *Hemipristis serra* (upper lateral teeth (MPM-1354)). (K–N) *Sphyrna* sp. (upper antero-lateral teeth (MPM-1381)). (O and P) Carcharhiniformes indet. vertebrae (MPM-1382). View: labial (B, D, F, H, J, L and M) and lingual (A, C, E, G, I, K and N).

**Figure 8:**
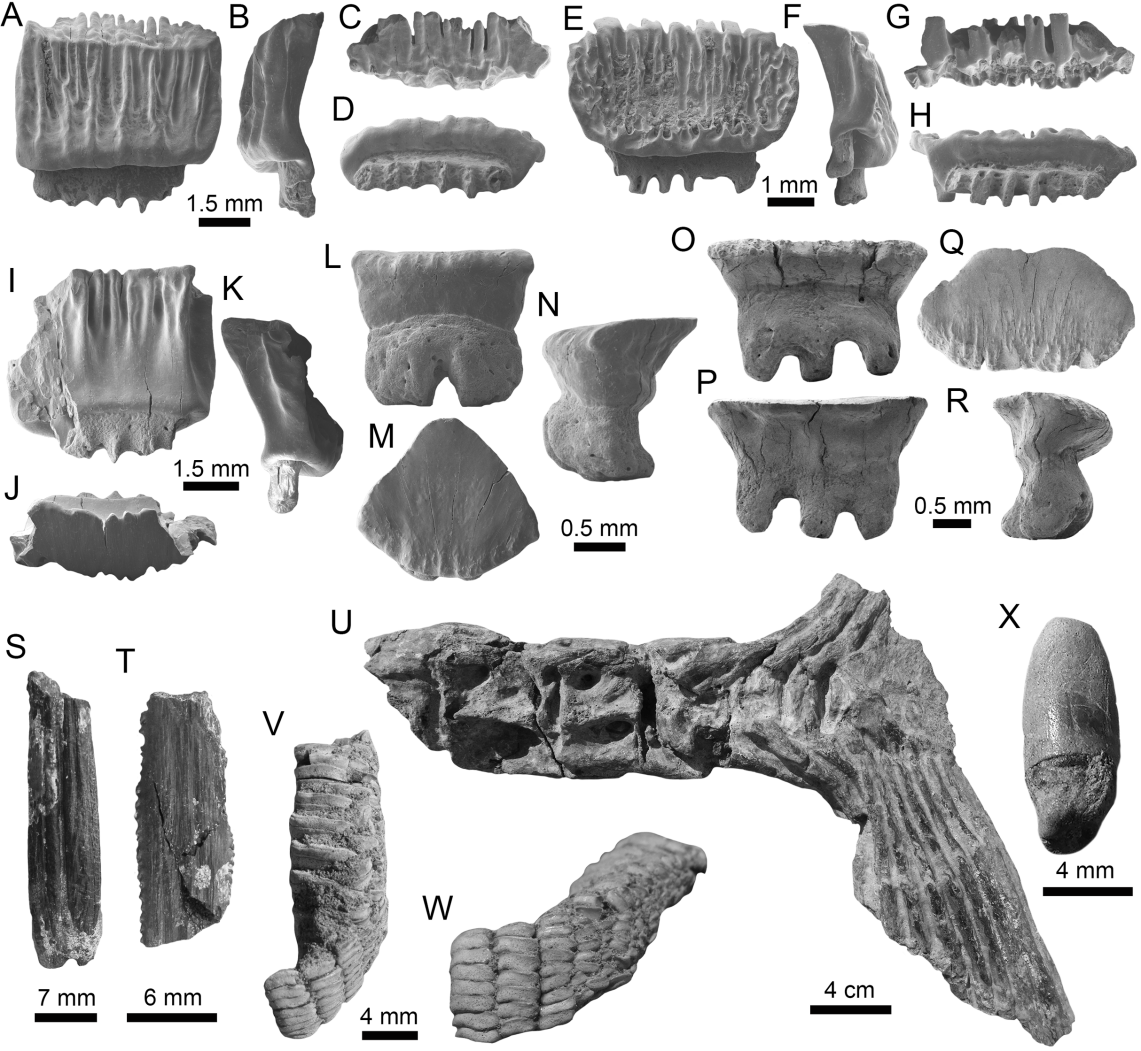
Myliobatiformes and Osteichthyes remains of the Montañita-Olón site (Dos Bocas Formation). (A–K) *Mobula fragilis* ((A–K): female antero-lateral teeth (MPM-1383)). (L–R) *Mobula* sp. ((L–R): female indet. position (MPM-1384)). (S and T) Myliobatiformes indet. (caudal spine fragments (MPM-1385)). (U) Caudal peduncle of *Eocoelopoma* sp. (MPM-1391). (V and W) Upper pharyngeal element of Hypsigenyini. (MPM-1392). (X) Isolated tooth of Labridae indet. (MPM-1393). View: labial (A, E, L, O, W), lingual (I, P), occlusal (C, G, J, M, Q, V), profile (B, F, K, N, R), basal (D, H), dorsal (T), ventral (S) and indet. (X), lateral (U).

The fossil elasmobranch fauna from the Montañita-Olón site consists of 424 cranial (teeth and rostral spines) and postcranial (vertebrae and caudal spines) elements (Figs. 2–8; Table 1; Data S1; Tables S1–S3); a few actinopterygian remains were also found. All specimens were collected in situ from different points along the Montañita-Olón cliff section (Fig. 2C), during several field trips conducted by the authors (JA, JDCB and FFA) and other collaborators between 2016 and 2018. The Ecuadorian Instituto Nacional de Patrimonio Cultural (INPC) excavation permit, Code: No 0039-DR5.INPC.2015 supported the field activities. The Montañita-Olón localities (Fig. 1) are located in a coastal cliff of around 800 m long, between the towns of Montañita and Olón (Santa Elena Province, Ecuador, coordinates: 1°48′9.3″S, 80°45′24,10W and 1°49′09.66″S, 80°45′30,50W). The fossil specimens are housed at the “Museo Paleontológico Megaterio” (MPM-) at the Universidad Estatal Peninsula de Santa Elena, Ecuador (Table S3). Photographs were taken with a Leica MZ16F multifocal stereomicroscope and Scanning Electronic Microscope (SEM) for small teeth. Tooth measurements including total height, width and length (see Fig. S1), were taken for the specimens and are listed in Table S1. Dental terminology and the systematics utilized herein follow Cappetta (2012). We identified all fossil elasmobranch remains to the lowest possible taxonomic level. Taxonomic identifications are based on literature review and comparative analysis between fossil and extant specimens from several collections including: the Fossil Vertebrate Section of the Museum für Naturkunde, Berlin, Germany (MB.Ma.); Natural History Museum of Basel (NMB), Switzerland; Natural History Museum of Vienna (NHMW), Austria; Paleontological collection of the Alcaldía del Municipio Urumaco (AMU-CURS); Paleontological collection of the Institut des Sciences de l’Evolution, University of Montpellier (UM), France; Palaeontological Institute and Museum at the University of Zurich (PIMUZ) and the René Kindlimann private collection with public access, Aathal, Switzerland; paleontological collections of the Mapuka Museum of Universidad del Norte (MUN), Barranquilla, Colombia.

**Table 1:** Elasmobranch paleodiversity of the Montañita-Olón site (Dos Bocas Formation).

Superorder	Order	Family	Genus	Taxon
Squalomorphii	Hexanchiformes	Heptranchidae	*Heptranchias*	*Heptranchias* cf. †H. howellii*
		Hexanchidae	*Hexanchus*	*Hexanchus* cf. *H*. griseus*
	Squaliformes	Centrophoridae	*Centrophorus*	*Centrophorus* cf. *C*. granulosus*
		Dalatiidae	*Dalatias*	*Dalatias* sp.*
	Echinorhiniformes	Echinorhinidae	*Echinorhinus*	cf. *Echinorhinus* sp.
			†*Paraechinorhinus*	†*Paraechinorhinus* cf. †*P*. barnesi*
	Pristiophoriformes	Pristiophoridae	*Pristiophorus*	*Pristiophorus* sp.
Galeomorphii	Orectolobiformes	Rhincodontidae	*Rhincodon*	*Rhincodon* sp.*
	Lamniformes	Lamnidae	*Isurus*	*Isurus* cf. I. oxyrinchus*
			Indet.	Indet.
		Mitsukurinidae	*Mitsukurina*	*Mitsukurina* cf. †*M*. lineata*
		Odontaspididae	*Carcharias*	*Carcharias* sp.
			*Odontaspis*	*Odontaspis* sp.*
		†Otodontidae	†*Otodus*	†*Otodus* (*Carcharocles*) cf. †O. angustidens*
			†*Parotodus*	†Parotodus benedenii*
			†*Megalolamna*	†Megalolamna paradoxodon*
		Alopiidae	*Alopias*	*Alopias* cf. †A. exigua*
				†Alopias latidens*
	Carcharhiniformes	Carcharhinidae	*Carcharhinus*	†Carcharhinus gibbesii*
			*Galeocerdo*	†Galeocerdo aduncus*
			†*Physogaleus*	†Physogaleus contortus*
		Hemigaleidae	*Hemipristis*	†*Hemipristis serra*
		Sphyrnidae	*Sphyrna*	*Sphyrna* sp.
		Indet.	Indet.	Indet.
Batomorphii	Myliobatiformes	Mobulidae	*Moluba*	†Mobula fragilis*
				*Mobula* sp. (this morphotype*)
		Indet.	Indet.	Indet.

**Note:**

* First fossil record in Ecuador.

An analysis of abundance for the Montañita-Olón site assemblage was carried out using percentages of specimens by family, genera and species. In addition, we performed a paleobathymetric analysis following the methodology of Nolf & Brzobohatý (1994), adapted to fossil sharks (Carrillo-Briceño et al., 2015a, 2016a, 2016b). A second paleobathymetric analysis following the methodology of Perez et al. (2017) was performed using R (R Core Team, 2019). This depth estimator uses weighted bootstrap analysis to estimate the depth distribution of the population sampled. To apply this technique, we calculated the mean for the total depth distribution range and the mean for the common distribution range for each taxon. A bootstrap was performed with the mean differences and using the relative abundance of each taxa as weight estimator of the mean. The data were resampled 10,000 times and plotted as a histogram that provides the mean depth. The 95% confidence interval was obtained using a percentile method. The raw data and script for the paleobathymetric analysis were included in Data S2. The average, minimum and maximum depth estimates were plotted in Fig. 10. The minimum and maximum depth are the lower and upper limits of the mean depth range in the distribution. For both analyses, we included only species/genera with closely related extant taxa. Extinct taxa without clear identification to the generic level were removed. We analyzed a total of 19 (out of 24) taxa that represent a total of 373 individuals for which ecological information is available based on closely related extant taxa. The bathymetric (depth range) and habitat preferences utilized for these analyses are available in Table S2, compilation following Compagno (1984a, 1984b), Compagno, Dando & Fowler (2005), Musick, Harbin & Compagno (2004), Ebert & Stehmann (2013) and the FishBase website (Froese & Pauly, 2019). We use the term “Tropical America” (Neotropics) to refer to the geographic area of the Western Hemisphere located between the Tropic of Cancer (23°27′ N) and the Tropic of Capricorn (23°7′ S). The ECP and the WCA are here referred to as the oceanic areas of Tropical America. The Eastern Pacific is referred to as the oceanic area from North America to the most southern point of South America.

**Figure 9:**
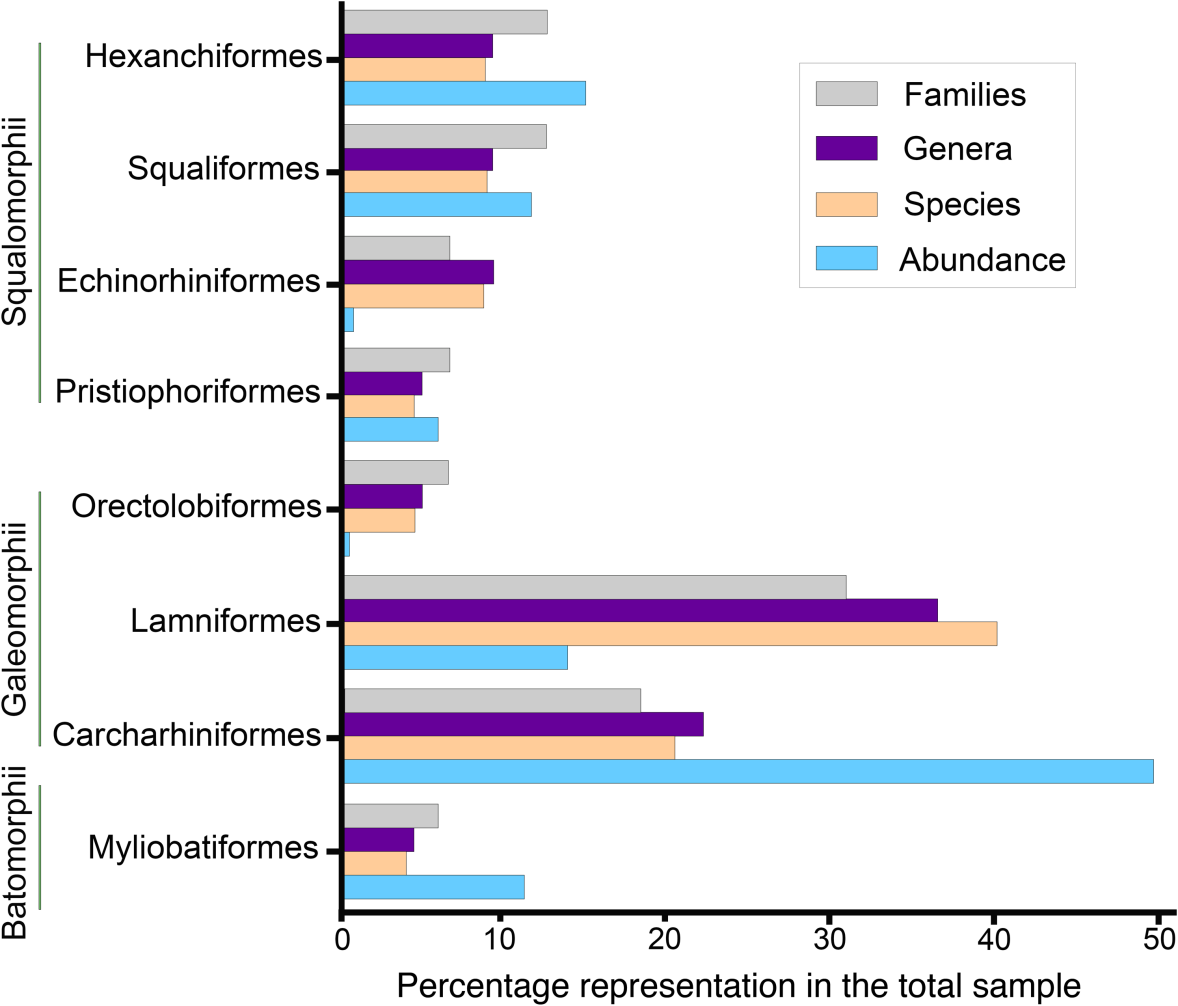
Elasmobranch paleodiversity of the Montañita-Olón site (Dos Bocas Formation).

## Results

### Elasmobranch paleodiversity

The elasmobranch assemblage described herein from the Montañita-Olón site comprises 27 taxa (including three of indeterminate taxonomy) of squalomorphs, galeomorphs and batoids (Figs. 3–8; Table 1; Tables S1 and S2). A general descriptive taxonomy of the dental elements of key species (first and rare records in Ecuador and Tropical America) is presented in Data S1.

Squalomorphs, with seven species, are the second most diverse and abundant group of sharks in the Montañita-Olón assemblage (Fig. 9; Table 1), and their remains are represented exclusively by isolated teeth (Table S1). The identified taxa include the hexanchiforms *Heptranchias* cf. *H*. *howellii* (Reed, 1946) (Figs. 3A–3H; Data S1) and *Hexanchus* cf. *H*. *griseus* (Bonnaterre, 1788) (Figs. 3I–3O; Data S1), the squaliforms *Centrophorus* cf. *C*. *granulosus* (Bloch & Schneider, 1801) (Figs. 3P–3W; Data S1) and *Dalatias* sp. (Figs. 3X and 3Y; Data S1), the echinorhiniforms cf. *Echinorhinus* sp. (Figs. 4A and 4B; Data S1), and *Paraechinorhinus* cf. *P*. *barnesi Welton in* Pfeil, 1983 (Figs. 4C and 4D; Data S1), and the pristiophoriform *Pristiophorus* sp. (Figs. 4E–4H). With the exception of *Pristiophorus* sp., all the above-mentioned species are reported for the first time in the fossil record of Ecuador. *Heptranchias* cf. *H. howellii*, *Centrophorus* cf. *C. granulosus*, *Dalatias* sp., cf. *Echinorhinus* sp. and *Paraechinorhinus* cf. *P. barnesi* are reported for the first time in the fossil record of the ECP.

Galeomorphs (isolated teeth as well as vertebral remains) represent the most diverse and abundant group in the elasmobranch assemblage from the Montañita-Olón site (Fig. 9). This group of sharks is characterized by a total of 15 species within 14 genera and nine families of Orectolobiformes, Lamniformes and Carcharhiniformes (Table 1). Lamniforms represent the most diverse group in the overall assemblage of the Montañita-Olón site. With 10 taxa, it includes: *Isurus* cf. *I*. *oxyrinchus* Rafinesque, 1810 (Figs. 4L–4S; Data S1), an indeterminate lamnid species (represented by and isolated tooth, see Figs. 6A–6C; Data S1), *Mitsukurina* cf. *M. lineata* (Probst, 1879) (Figs. 4T–4Z; Data S1), *Carcharias* sp. (Figs. 5A and 5B; Data S1), *Odontaspis* sp. (Figs. 5C–5K; Data S1), *Otodus (Carcharocles)* cf. *O. angustidens* (Agassiz, 1833–1843) (Figs. 5L–5Q; Data S1), *Parotodus benedenii* (Le Hon, 1871) (Figs. 5R–5T; Data S1), *Megalolamna paradoxodon* (Shimada et al., 2017) (Figs. 5U–5X; Data S1), and *Alopias* cf. *A. exigua* (Probst, 1879) (Figs. 6D–6H). Carchariniforms are represented by *Carcharhinus gibbesii* (Woodward, 1889) (Figs. 6N–6S; Data S1), the most abundant taxon in the assemblage (Table S2), as well as *Galeocerdo aduncus* (Agassiz, 1833–1843) (Figs. 6T–6W), *Physogaleus contortus* (Gibbes, 1849) (Figs. 7A–7F), *Hemipristis serra* (Agassiz, 1833–1843) (Figs. 7G–7J), and *Sphyrna* sp. (Figs. 7K–7N). A group of 25 semi articulated vertebrae (Figs. 7O and 7P) was also collected in the Montañita-Olón site inside a concretion (Fig. 2C); however, due to the lack of diagnostic elements, a more accurate identification than “Carcharhiniformes indet.” is not possible. The presence of an incomplete tooth of the whale shark *Rhincodon* sp. (Figs. 4I–4K; Data S1) represents the only record of an orectolobiform shark in the Montañita-Olón site. With the exception of *Carcharias* sp. and *H. serra*, all the above-mentioned galeomorph species are reported for the first time in the fossil record of Ecuador. *Rhincodon* sp., *Mitsukurina* cf. *M. lineata*, *Odontaspis* sp., *Otodus (Carcharocles)* cf. *O. angustidens*, *Alopias* cf. *A. exigua*, *Alopias latidens* and *Carcharhinus gibbesii* are reported for the first time in the fossil record of the ECP.

The batoids, with only one genus and two species, are the least diverse group from the Montañita-Olón assemblage (Fig. 9; Table 1). Only a few isolated teeth of the mobulids *Mobula fragilis* (Cappetta, 1970) (Figs. 8A–8K; Data S1) and *Mobula* sp. (Figs. 8L–8R; Data S1) are herein reported. *Mobula fragilis* is here reported for the first time in the fossil record of Ecuador and the ECP. Although some isolated mobulid teeth have been reported before from Neogene deposits of the Central Eastern Pacific of Ecuador (Carrillo-Briceño, Aguilera & Rodríguez, 2014) and Panama (Carrillo-Briceño et al., 2018), the morphological pattern of the *Mobula* sp. specimens from the Montañita-Olón site has not been recognized before in the ECP. In addition, two eroded, broken and non-diagnostic caudal spine fragments (Figs. 8S and 8T) are referred herein to Myliobatiformes indet.

### Paleobathymetric analysis

Two paleobathymetric methods were applied to the elasmobranch fauna from the Montañita-Olón site: (1) an adaptation of Nolf’s method which calculates the percentage of species that share an assigned depth range (Nolf & Brzobohatý, 1994) and (2) the weighted method (Perez et al., 2017) by resampling the mean differences and using the relative abundance as weight estimator of the mean. Nineteen of the 24 total taxa, which were represented by 373 specimens (88.39% of total studied material), were included in the analysis. We used only taxa that have comparable modern analogs that allow us to estimate their depth ranges (Carrillo-Briceño et al., 2015a). This allows us to obtain more accurate estimations. For taxa without modern representatives, their presence is assumed in the estimated depth range.

**Figure 10:**
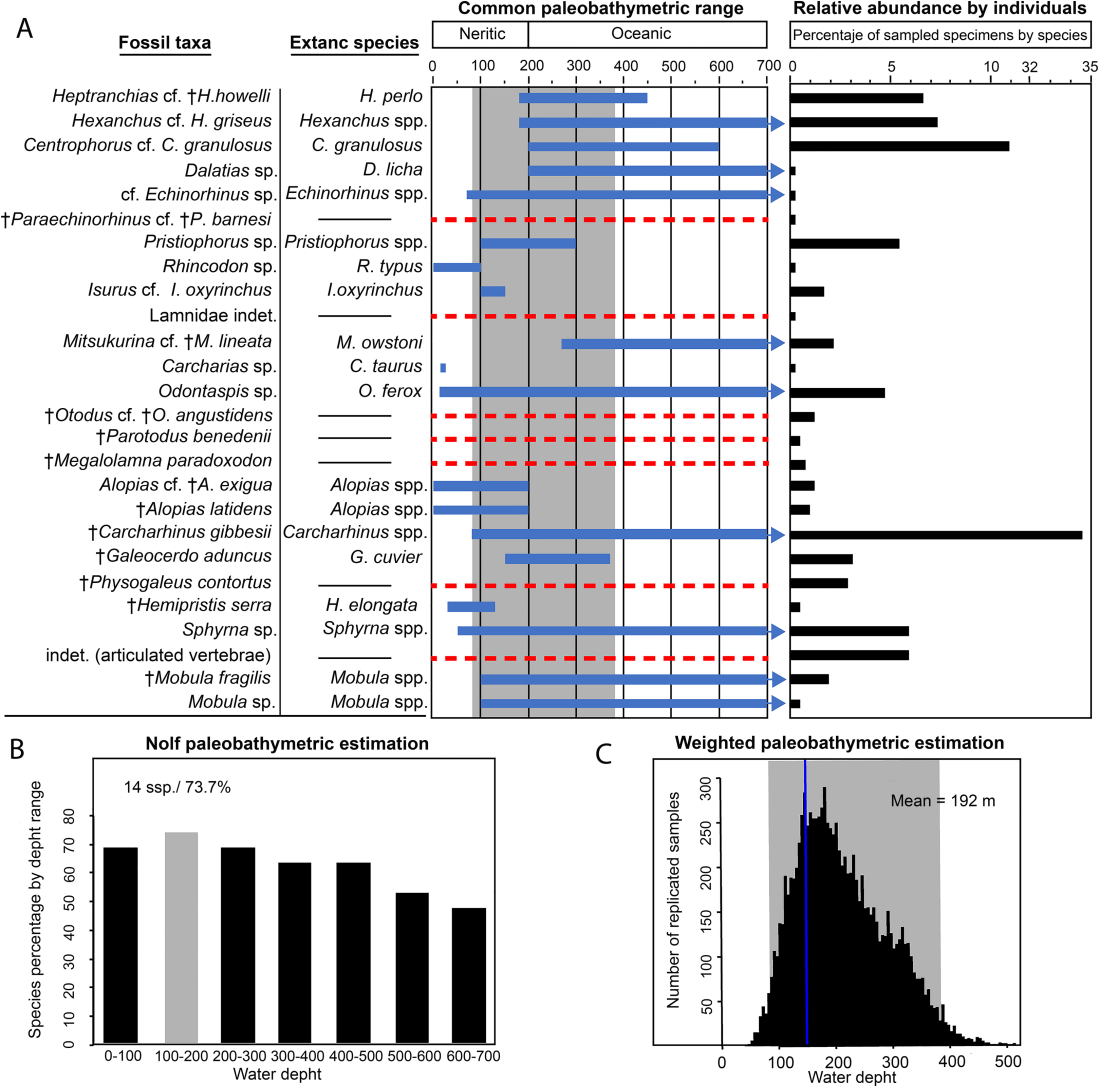
Paleobathymetric estimation for the Montañita-Olón site (Dos Bocas Formation) elasmobranch taxa, their relative abundance and paleodiversity. (A) Blue lines indicate the common paleobathymetric range for each taxa, arrows indicate that the bathymetric range is greater, a dashed red line indicates that there is not information for the taxa and that was removed from the analysis. The gray shadow indicates the 95% confidence intervals for the more probable depth range for this assemblage. (B) For the Nolf technique, 73.7% of the studied taxa are represented in a depth range of 100–200 m. (C) The weighted paleobathymetric estimation indicate a mean of 192 m after resampling with 10,000 simulations. The *y*-axis is shortened between 11 and 32 to improve the data visualization in the relative abundance graphic.

Both results were comparable. For the Nolf technique, 73.7% of the studied taxa are represented in a depth range of 100–200 m (Fig. 10B). For the weighted paleobathymetry, a range between 87.8 and 378.0 m with a mean of 192 m was estimated (Fig. 10C). Our results suggest that the fossils were most likely deposited in an outer neritic (open shelf) environment. Of these two applied techniques, the weighted method provides more precision, but the Nolf method allows a better visualization of the depth distribution for each taxa in the assemblage (Fig. 10). The results obtained using these two techniques, improves the data interpretation in our study.

## Discussion

### Diversity composition and biostratigraphy significance

Elasmobranchs from the Oligocene and early Miocene have not been reported from Ecuador, and their fossil record has been restricted to younger strata (Longbottom, 1979; Carrillo-Briceño, Aguilera & Rodríguez, 2014). The elasmobranch assemblage described here from the Montañita-Olón site includes at least 27 taxa (Table 1). This represents the most diverse elasmobranch assemblage known from Ecuador and for the Oligocene–Miocene boundary of Tropical America. The assemblage includes 13 extinct taxa (e.g., *Heptranchias* cf. *H*. *howellii*, *Paraechinorhinus* cf. *P*. *barnesi*, *Mitsukurina* cf. *M*. *lineata*, *Otodus* (*Carcharocles*) cf. *O*. *angustidens*, *P*. *benedenii*, *M*. *paradoxodon*, *Alopias* cf. *A*. *exigua*, *A*. *latidens*, *C. gibbesii*, *G. aduncus*, *P*. *contortus*, *H*. *serra* and *M*. *fragilis*) with a worldwide paleodistribution (Cappetta, 2012; Cappetta, Gregorová & Adnet, 2016; Szabó & Kocsis, 2016a; Shimada et al., 2017). From the overall elasmobranch assemblage at Montañita-Olón site, 19 species are reported here for the first time in the fossil record of Ecuador (Table 1). Ten species, including the squalomorphs *Heptranchias* cf. *H*. *howellii*, *Centrophorus* cf. *C*. *granulosus*, *Dalatias* sp., cf. *Echinorhinus* sp., the galeomorphs *Rhincodon* sp., *Mitsukurina* cf. *M*. *lineata*, *Odontaspis* sp., *Carcharhinus gibbesii* and the batoids *Mobula fragilis* and *Mobula* sp., are reported for the first time in the fossil record of the ECP. Records of *Paraechinorhinus* cf. *P*. *barnesi*, *Otodus* (*Carcharocles*) cf. *O*. *angustidens*, *Alopias* cf. *A*. *exigua*, *A*. *latidens*, are reported for the first time in the fossil record of South America. The presence of *Rhincodon* sp. from Ecuador, together with the record from the late Oligocene (Chattian) of Eastern USA (Cicimurri & Knight, 2009), represent the oldest records for this taxon. The *Heptranchias* cf. *H*. *howellii* specimens from the Montañita-Olón site (see Data S1) clearly resemble teeth of *H*. *howellii* from the Oligocene of North America (Welton, 1974), early Miocene of Colombia (Carrillo-Briceño et al., 2016b) and other regions (Cappetta, Gregorová & Adnet, 2016). The presence of *Paraechinorhinus* cf. *P*. *barnesi* in the Oligocene–Miocene of Ecuador increases its paleobiogeographic distribution and represents the oldest record of the genus in the Americas, as *P*. *barnesi* was known only from the middle Miocene of California (USA) (Pfeil, 1983; Cappetta, 2012).

As mentioned above in the results section, mobulid teeth identified as *Mobula* sp. have been reported before in the ECP from the Middle Miocene-early Pliocene of Ecuador (Carrillo-Briceño, Aguilera & Rodríguez, 2014, fig. 5) and late Miocene-Pliocene of Panama (see Perez et al., 2017; Carrillo-Briceño et al., 2018, table S6 and references therein). However, the morphological pattern of the *Mobula* sp. specimens from the Montañita-Olón site (Figs. 8L–8R) differ from those teeth reported from Ecuador and Panama. The two *Mobula* sp. specimens referred to here (MPM-1384), as well as those specimens illustrated by Carrillo-Briceño et al. (2016a, fig. 11.19*–*21*)* from the early Miocene of Venezuela, resemble teeth of the living species *Mobula munkiana Notarbartolo* Di Sciara (1987) (Adnet et al., 2012, fig. 4). Nevertheless, due to the scarcity of fossil and comparative material, a more accurate specific identification is not possible at this time. The batoids are the less diverse group from the Montañita-Olón assemblage with only benthopelagic representatives (Table S2). The absence of other batoids, especially those associated benthonic habitats, could likely be a result of bias in sampling. However, the benthonic batoids could also be associated with the ecological and environmental conditions that prevailed during the deposition of the Dos Bocas Formation, (e.g., Cappetta, Gregorová & Adnet, 2016).

To date, the elasmobranch assemblage from the Montañita-Olón site is the only one known from Oligocene–Miocene boundary of the ECP. Other Oligocene elasmobranchs from the region are unknown, and only a few early Miocene assemblages are restricted to southern Peru (Landini et al., 2019). The elasmobranch assemblage reported by Landini et al. (2019) from the Chilcatay Formation which is characterized by 22 taxa, clearly contains faunal differences in comparison with those from the Montañita-Olón site, where only a few galeomorphs (e.g., *Isurus oxyrinchus*, *Carcharias*, *P*. *benedenii*, *M*. *paradoxodon*, *G. aduncus*, *P*. *contortus* and *H*. *serra*) were present in both geological units. A few early Miocene assemblages from the southernmost areas of the Eastern Pacific in Chile have also been described (Villafaña & Rivadeneira, 2018; Villafaña et al., 2019). As in the Montañita-Olón site, early Miocene assemblages from Chile include taxa such as *Carcharias*, *Odontaspis*, *Isurus* (Villafaña et al., 2019), including a new species of sawshark †*Pristiophorus humboldti*, Villafaña et al. (2019) from the Navidad Formation. The presence of *Pristiophorus* sp. in the Montañita-Olón site represents the oldest record for this taxon in the ECP, although the taxon has been reported from the Eocene of southernmost Magallanes Region (Otero et al., 2013). The isolated rostral spines reported here show the typical characters described for the genus *Pristiophorus* from the Eastern Pacific (Carrillo-Briceño et al., 2013; Staig et al., 2015; Villafaña et al., 2019). However, the use of rostral spines is not recommended for species identification due to their high variability and insufficiency as a diagnostic character (Underwood & Schlögl, 2013; Engelbrecht et al., 2017; Villafaña et al., 2019). By contrast, the teeth display enough characters to assign specimens to lower taxonomic levels. Therefore, we prefer to identify to the genus level until more material is available. In addition, two coprolites with abundant bony fish remains and some isolated rostral spines of *Pristiophorus* were found in the Montañita-Olón site (Fig. S2). Although it is difficult to identify the possible producer of these coprolites, there is no doubt that sawfishes were part of the predator’s diet.

Like *Pristiophorus*, other elasmobranch taxa such as *Dalatias*, *Carcharias*, and *Isogomphodon*, were at the end of the Neogene regionally extirpated from the Eastern Pacific, but with extant representatives still inhabiting the Western Atlantic (Carrillo-Briceño et al., 2018). The presence of *Dalatias* sp. in the Montañita-Olón site is the oldest record of this taxon in the Eastern Pacific. This record from Ecuador together with the one known from the late Miocene–Pliocene of the Atacama Region in Chile (Villafaña & Rivadeneira, 2018), suggest that *Dalatias* was present in the Eastern Pacific from the late Oligocene to the end of the Neogene, when it became regionally extinct. The presence of *Mitsukurina* cf. *M*. *lineata* in the Montañita-Olón site also suggests the extirpation of the genus from the Eastern Pacific (see Carrillo-Briceño et al., 2018, table S4).

A late Oligocene–early Miocene age has been suggested for the Dos Bocas Formation on the basis of radiometric dating (Witt et al., 2019). According to Witt et al. (2019), the U-Pb zircon dating for the Montañita-Olón outcrop in the Montañita area (Fig. 2B) yielded an age of 23.5 ± 0.4 Ma, with a younger cluster average of 22.9 ± 0.6 Ma. These results agree with a late Oligocene age proposed for the unit using faunal composition (Olsson, 1931; Bristow, 1975; Tanaka et al., 2017). In addition, the age of the Dos Bocas Formation (Montañita-Olón site) proposed here is also supported by the presence of the typical Oligocene megatooth species *Otodus* (*Carcharocles*) cf. *O*. *angustidens* from other localities around the world (Baut & Génault, 1999; Gottfried & Fordyce, 2001; Reinecke, Stapf & Raisch, 2001; Szabó & Kocsis, 2016b).

### Paleoenvironmental inferences

A shallow protected environment has been suggested as the most plausible depositional environment for the Dos Bocas Formation (see Tanaka et al., 2017; Witt et al., 2019, and references therein). The base of the fossiliferous outcrop consists of massive, moderately sorted, fine to medium-grained sandstone with angular quartz-feldspathic clasts and probably glauconite rounded green grains (Fig. 2). The matrix is micritic and volcanogenic, possibly bentonitic. According to Tanaka et al. (2017), bedding is massive to indistinct, suggesting little influence by traction currents or storm waves, and in turn implying a quiet setting; estuarine or mid-shelf is possible. Witt et al. (2019, fig. 8A*)* reported for the outcrops of the Dos Bocas Formation in the Montañita-Olón area, local large arthropod burrows and some evidence of patch reefs of bivalves and worms, suggesting that these beds were deposited in a protected shallow-water environment, likely under a period of strong tectonic deformation. Nevertheless, arthropod burrows are not exclusively from shallow water environments, and our field observations suggest that the thick layer of large arthropod burrows and the probable evidence of patch reefs with fragmentary mollusks reported by Witt et al. (2019) for the Montañita-Olón area are represented towards the top of the section (Fig. 2A). In contrast, micropaleontological evidence suggests for the Dos Bocas Formations, by Ordoñez, Jiménez & Suárez (2006) and Witt et al. (2019, fig. 3C*)*, an upper platform environment. In our bathymetric analysis of the Montañita-Olón site (Dos Bocas Formation), 73.7% of the studied elasmobranch taxa are represented in a depth range of 100–200 m (Fig. 10). For the weighted paleobathymetry, a range between 87.8 and 378.0 m with a mean of 192 m was estimated. Our results suggest that the fossils were most likely deposited in an outer neritic (open shelf) environment, which coincides with the upper platform environment suggested by Witt et al. (2019, fig. 3C*)*. Additional support for this estimation is derived from the presence of the benthopelagic squalomorph sharks *Heptranchias* cf. *H*. *howellii*, *Hexanchus* cf. *H*. *griseus*, *Centrophorus* cf. *C*. *granulosus*, *Dalatias* sp. and cf. *Echinorhinus* sp., whose extant representatives usually prefer deep-water environments near the continental slope (see Table S2). The extinct *Heptranchias* cf. *H*. *howellii* has been reported from other localities of the Americas and Europe as a species with deep environment preferences (Cappetta, Gregorová & Adnet, 2016; Carrillo-Briceño et al., 2016b and references there in). *Paraechinorhinus* cf. *P*. *barnesi* is another extinct taxon associated with deep-water paleoenvironments (Welton, 1979; Pfeil, 1983). In addition, the presence of “Goblin shark” *Mitsukurina* cf. *M*. *lineata* in the Montañita-Olón assemblage could support our bathymetric estimation. The extant *Mitsukurina* bottom-dwelling species is usually living in deep waters, on the outer continental shelves and upper slopes (Compagno, 1984a; Cappetta, Gregorová & Adnet, 2016); although it should not be ruled out that the species could also have occasional visits to the neritic areas (Ebert & Stehmann, 2013). As an example, an isolated tooth assigned to *Mitsukurina* was referred from an infralittoral environment during the early Miocene of Costa Rica (Laurito et al., 2014).

Some of the benthopelagic and pelagic galeomorph taxa of the Montañita-Olón assemblage (Table S2) have been associated with both coastal and open-water environments in the fossil record, as well as for their living representatives (Laurito, 1999; Aguilera, 2010; Cappetta, 2012; Compagno, 1984b, Purdy et al., 2001; Compagno, Dando & Fowler, 2005; Ebert & Stehmann, 2013; Carrillo-Briceño et al., 2013, 2015b, 2016a, 2018, 2019; Perez et al., 2017; Landini et al., 2019, and references there in). Taxa such as *Isurus* cf. *I*. *oxyrinchus*, *Carcharias* sp., *Odontaspis* sp., *Otodus* (*Carcharocles*) cf. *O*. *angustidens*, *Parotodus benedenii*, *M. paradoxodon*, *Alopias* cf. *A*. *exigua*, *G*. *aduncus*, *P*. *contortus* and *H*. *serra* have been reported with a wide global distribution during the Cenozoic (Gottfried & Fordyce, 2001; Cappetta, 2012; Reinecke et al., 2014; Carrillo-Briceño, Aguilera & Rodríguez, 2014; Carrillo-Briceño et al., 2015a), suggesting significant distances over oceanic basins and wide environmental ranges. The extinct *Carcharhinus gibbesii*, with 146 isolated teeth in the total sample, is the most abundant taxon in the elasmobranch assemblage from the Montañita-Olón site. Like the above-mentioned sharks, *C. gibbesii* was an oceanodromous species with a wide distribution in North America, Europe and Tropical America during the Oligocene–early Miocene (Cicimurri & Knight, 2009; Reinecke et al., 2014; Carrillo-Briceño et al., 2016b, 2019). In the early Miocene of Colombia, *C. gibbesii* was reported in both shallow and deep-water environments (Carrillo-Briceño et al., 2016b, 2019).

Other vertebrates reported for the Montañita-Olón outcrops include the dolphin *Urkudelphis chawpipacha* Tanaka et al., 2017, and a Pan-Cheloniidae sea turtle (Cadena, Abella & Gregori, 2018). However, no environmental conditions were inferred on the basis of these taxa. Bony fishes, including the tail of a Scombridae (Fig. 8U), and dental battery and isolated tooth of Labridae (Figs. 8V–8X), were collected in the Montañita-Olón outcrops (Fig. 2C). The caudal peduncle of Scombridae in the Montañita-Olón is assigned to †*Eocoelopoma* Woodward, 1901 by having preural vertebrae 2–4 abruptly shortened and a hypural plate formed by hypurals 1–4 (-5) (Monsch & Bannikov, 2011). *Eocoelopoma* is a primitive scombrid closely related to the Sardini + Thunnini clade and cannot be confused with Thunnini because the reduction of the preural vertebrae in this tribe is extremely pronounced (Monsch & Bannikov, 2011). The genus *Eocoelopoma* is known from the Paleocene of Turkmenistan, early Eocene of England, and from Equatorial Africa without specific age. This report for the Montañita-Olón represents the first record of the genus for Tropical America (Bannikov, 1985; Monsch, 2004; Monsch & Bannikov, 2011). The presence of *Eocoelopoma* sp. in the Montañita-Olón locality suggests open to oceanic marine environments, due to the paleoenvironmental inferences suggested in other localities where this taxon was reported (Friedman et al., 2016). The caudal skeleton of *Eocoelopoma* presents an typical interlocking of vertebrae, which are well adapted to a pelagic mode of life (Fierstine & Walters, 1968). A second specimen from the Montañita-Olón locality represented by upper pharyngeal bones with phyllodont condition, where the teeth are developed in distinct stacks, is diagnostic of Hypsigenyini (Bellwood et al., 2019), and the multiple oblique tooth rows (Figs. 8V and 8W) are diagnostic characters for †*Trigonodon* (Bellwood et al., 2019). Hypsigenyini are restricted to the Miocene-Pliocene of Europe and Africa (Schultz & Bellwood, 2004; Bellwood et al., 2009), and *Trigonodon* in only known from the Miocene of Europe by the type species †*Trigonodon* oweni Sismonda in Michelotti (1847). Although *Trigonodon* has been referred from the early Miocene of Costa Rica (Laurito et al., 2014), a future detailed taxonomic revision for our specimens from Ecuador and those from Costa Rica could help give new insights on the taxonomy of these fossil Hypsigenyini fishes from Tropical America. According to Schultz & Bellwood (2004), *Trigonodon* was a shallow-water fish possibly living primarily on coral reefs and associated hard-grounds. The presence of shallow-water components in the Montañita-Olón site could be explained by elements being washed into deeper water by turbidity currents or slumping (Vialle, Adnet & Cappetta, 2011).

## Conclusions

We report 27 elasmobranch taxa, of which 19 are new fossil records for Ecuador, 10 new records for the Central Eastern Pacific and four new records for South America. This elasmobranch fossil assemblage represents the most diverse known from Ecuador and for the Oligocene–Miocene boundary of Tropical America. The elasmobranch habitat preferences and paleobathymetric analyses support the hypothesis that the Montañita-Olón site was likely deposited in an outer neritic (open shelf) environment. Future work and new findings could help improve and refine our interpretations. The assemblage from the Montañita-Olón site increases the fossil record of the region and represents a critical window into marine tropical vertebrate faunas in the ECP during the OMT, a critical moment in the evolutionary history of the marine biota of Tropical America.

## Supplemental Information

10.7717/peerj.9051/supp-1 Supplemental Information 1 Descriptive taxonomy of key elasmobranch species present in the Montañita-Olón site (Dos Bocas Formation).

10.7717/peerj.9051/supp-2 Supplemental Information 2 Raw data and script for the paleobathymetric analysis.

10.7717/peerj.9051/supp-3 Supplemental Information 3 Elasmobranchs of the Montañita-Olón site (Dos Bocas Formation) and their record per jaw position and tooth measurements. Abbreviations: indeterminate (Indet.).

10.7717/peerj.9051/supp-4 Supplemental Information 4 Bathymetric preferences of the Montañita-Olón site (Dos Bocas Formation) elasmobranch taxa, based on the biology of their extant relatives. Abbreviations: minimun (Mn), maximun (Mx), meters (m) and indeterminate (Indet.). Lifestyle: Benthic (B), Benthopelagic (Bp); Pelagic (P). Preferred Habitat Neritic (N), Oceanic (O), Sublitoral (S), Bathyal (Bt), Abysal (Ab), Hadal (Ha), Epipelagic (E), Mesopelagic (M), Bathypelagic (Bp).

10.7717/peerj.9051/supp-5 Supplemental Information 5 Referred fossil specimens from the Montañita-Olón site (Dos Bocas Formation) and their collection numbers.

10.7717/peerj.9051/supp-6 Supplemental Information 6 Tooth measurements.

10.7717/peerj.9051/supp-7 Supplemental Information 7 Coprolites from the Montañita-Olón site (Dos Bocas Formation). A. MPM-1389 and B. MPM-1390) with isolated rostral teeth of *Pristiophorus* sp. and indet. bony fish remains.

## Additional Information and Declarations

### Field Study Permissions

The following information was supplied relating to field study approvals (i.e., approving body and any reference numbers): Ecuadorian Instituto Nacional de Patrimonio Cultural (INPC) approved this field study (Excavation permit, Code: No 0039-DR5.INPC.2015).

### Data Availability

The following information was supplied regarding data availability: Raw data and script are available in Data S2. All the fossil specimens described here are available at the “Museo Paleontológico Megaterio” (MPM-) at the Universidad Estatal Peninsula de Santa Elena, Ecuador. Every specimen or group of specimens is assigned with a catalog number using the acronyms “MPM-”. Data on fossil specimens with their respective catalog numbers are available in Table S3.
