# Supplementary material for: Diversity and paleoenvironmental implications of an elasmobranch assemblage from the Oligocene–Miocene boundary of Ecuador
Source: PeerJ. 2020 Apr 29;8:e9051. doi: 10.7717/peerj.9051 (PMC7195833; doi:10.7717/peerj.9051)
Supplement: Supplemental Information 1 [file peerj-08-9051-s001.docx]

**Systematic paleontology**

Squalomorphii *Compagno, 1973*

Hexanchiformes *Buen, 1926*

Heptranchidae *Barnard, 1925*

*Heptranchias* *Rafinesque, 1810*

*Heptranchias* cf. †*H*. *howellii* (*Reed, 1946*)

(Fig. 3A-H)

Twenty-eight isolated teeth (Table S1) correspond to *Heptranchias* cf. *H*. *howellii.* Only few lower lateral teeth are complete, being elongate, strongly compressed labiolingually, with rectangular-shape root and with a well-developed smooth and distally inclined acrocone cusplets (Fig. 3F). The acrocone is larger and well differentiated from distal cusplets, of which up to six can be counted in the largest and most complete specimens. Between two and five small and well differentiated mesial denticles can be noticed. The presence of more than two mesial denticles at the base of the main cusp clearly differentiate *H*. *howellii* from the recent and other extinct *Heptranchias* species (*Welton, 1974*; *Cappetta, Gregorová* & *Adnet, 2016*). Upper teeth have a well-developed narrow cusp with sigmoidal contour and up to two distal cusplets in the lateral teeth (Figs. 3G and 3H). These specimens from the Montañita-Olón site clearly resemble those teeth of *H*. *howellii* from Norte America and other regions (see *Welton, 1974*; *Cappetta, Gregorová* & *Adnet, 2016*, *Carrillo-Briceño et al., 2016b*).

Hexanchidae *Gray, 1851*

*Hexanchus* *Rafinesque, 1810*

*Hexanchus* cf. *H*. *griseus* (*Bonnaterre, 1788*)

(Fig. 3I-O)

Thirty-one teeth (Table S1) are herein assigned to *Hexanchus* cf. *H*. *griseus*. Lower teeth are compressed labiolingually with a high root (rectangular in shape), a well-defined acrocone and up to 10 distal cusplets in the largest specimen; a well-developed small mesial serration is present (Fig. 3I-M). Upper teeth show a slightly sigmoidal and distally inclined acrone followed by up to two distal cusplets in the lateral teeth (Figs. 3N and 3O). According to *Cappetta (2012)*, the teeth of *Hexanchus* are more mesio-distally elongated than in *Notorynchus* as the specimens described here. Additionally, the acrocone is slightly bigger than the distal cusplets in *H. griseus* whereas in *H. nakamurai* its remarkable bigger. Therefore, the material described here can be unambiguously identified at generic level and show similar characters with those teeth of *H. griseus* from the late Miocene-Pliocene of Costa Rica and Chile (*Long, 1993*; *Laurito, 1999*; *Carrillo-Briceño et al., 2013*; *Staig et al., 2015*).

Squaliformes *Goodrich, 1909*

Centrophoridae *Bleeker, 1859*

*Centrophorus* *Müller & Henle, 1837*

*Centrophorus* cf. *C. granulosus* (*Bloch & Schneider, 1801*)

(Fig. 3P-W)

Forty-six teeth (Table S1) were identified as *Centrophorus* cf. *granulosus*. The lower antero-lateral teeth are labio-lingually compressed with a broad and distally inclined cusp (Fig. 3S-W). The mesial cutting edge is slightly convex and serrated. The distal heel is high and convex without any serration. The apron is long and broader at its base in labial view. In lingual view, a short uvula and deep infundibulum can be observed in some teeth. The root is high and show several small foramina at it distal and mesial region. In upper antero-lateral teeth, the cusp is higher than broad (Figs. 3V and 3W). The mesial cutting edge is slightly serrated and concave. The distal heel is high and strongly convex without any serration. The uvula is very short, showing a large and deep infundibulum at its base. The presence of slightly serrated mesial cutting edges and the absence of ornamentation on the uvula are the diagnostic characters of the living species *C. granulosus* (*Vialle, Adnet & Cappetta 2011*). The material described here matched with those teeth of *C. granulosus* reported from the late Miocene of Panamá (*Carrillo-Briceño et al., 2015*).

Dalatiidae *Gray, 1851*

*Dalatias* *Rafinesque, 1810*

*Dalatias* sp. (*Bonnaterre, 1788*)

(Figs. 3X and 3Y)

A single lower tooth (Table S1) was identified as *Dalatias* sp. The lower tooth is higher than broad and labio-lingually compressed (Figs. 3X and 3Y). The crown is triangular and slightly leans towards the rear. The cutting edges are strongly serrated that are directed upwards. The lower mesial part of the tooth is missing. The distal heel is convex and weakly serrated. The preserved root is high with a convex distal edge. A shallow medio-lingual foramen is present. The labial apron and the lingual channel-shaped depression are not preserved. The genus has been reported from the early Miocene of Colombia and late Miocene of Panamá (as *D. licha*, *Carrillo-Briceño et al., 2015*, *2016b*). However, due to the much-abraded condition of the tooth and their incompleteness it is not possible to identify at specific level.

Echinorhiniformes *Buen, 1926*

Echinorhinidae *Gill, 1862*

*Echinorhinus Blainville, 1816*

cf. *Echinorhinus* sp.

(Figs. 4A and 4B)

A single incomplete antero-lateral tooth (Table S1) which is broader than high with a long and distally inclined cusp (Figs. 4A and 4B). The mesial cutting edge is weakly serrated and slightly concave at its base whereas the upper part is convex. An incomplete root but seemingly very low is preserved. Although the tooth is not well preserved, the presence of low and distally inclined cusp can be used as a diagnostic character of the genus *Echinorhinus* (*Cappetta, 2012*). Both the genus *Echinorhinus* and *Paraechinorhinus* coexisted often together in their life history. Our decision to determine this tooth fragment to cf. *Echinorhinus* bases mainly on the fact that the main cusp is distinctly more slender and more bent at the lower side of the crown, contrary to what is known of *Paraechinorhinus* (*Pfeil, 1983*). Besides that, the crown of the specimen shows a slight persistence at its base, a feature also not being typical to teeth of *Paraechinorhinus*. Additionally the tooth serration is more irregular in *Echinorhinus* instead being smoother in *Paraechinorhinus*, at least at the distal side of the crown. In spite of this, it is not fully impossible that this tooth fits into the variation of *Paraechinorhinus*; more material will be necessary for further studies.

†*Paraechinorhinu*s *Welton in Pfeil, 1983*

†*Paraechinorhinus* cf. †*P. barnesi* *Welton in Pfeil, 1983*

(Figs. 4C and 4D)

A single tooth (Table S1) was identified as *Paraechinorhinus* cf. *P. barnesi.* The presumed antero-lateral tooth displays a short and raised cusp; the apex is missing (Figs 4C and 4D). The mesial cutting edge is convex at its base and smooth whereas it is slightly concave and weakly serrated at its upper part. The distal heel is short and apically convex without any serrations. The root is moderately high and rectangle-shaped; the mesial part is missing. According to the original description of *Welton* (*1979*), the dental characters described here and the absence of mesial and distal cusplets are the diagnostic character of *P. barnesi.* Therefore, the specimen described here matched with those teeth reported from the middle Miocene of North America (*Pfeil, 1983*).

Galeomorphii *Compagno, 1973*

Orectolobiformes *Applegate, 1972*

Rhincodontidae *Garman, 1913*

*Rhincodon Smith, 1829*

*Rhincodon* sp.

(Figs. 4I-K)

A single tooth (Table S1) was identified as *Rhincodon* sp. The upper antero-lateral tooth displays a slender and erect cusp, which is slightly lingually oriented (Figs. 4I-K). The preserved lingual and labial faces of the crown are convex; distal part of the lingual face is missing (Fig. 3I). The enameloid surface is smooth. The apron is bulky and rounded at its end. The root is high, bulky with a strong lingual protuberance, bilobed and with a convex basal face (Fig. 4K). Several foramina are present in profile view (Fig. 4J). The tooth described here resembles of those teeth reported from the late Oligocene USA (*Cicimurri & Knight, 2009*) and late Miocene-early Pliocene of Costa Rica (*Laurito, 1999*).

Lamniformes *Berg, 1937*

Mitsukurinidae *Jordan, 1898*

*Mitsukurina Jordan, 1898*

*Mitsukurina* cf. †*M. lineata* (*Probst, 1879*)

(Fig. 4T-Z)

Nine teeth (Table S1) were identified as *Mitsukurina* cf. *M. lineata.* The lower anterior teeth show a very high and slender cusp with sigmoidal profile (Fig. 4T-V). The cutting edges are smooth, reaching the base of the crown. The root is high with long and separated lobes, forming an angel of about 70º (Fig. 4T). The median furrow is narrow and shallow, present in anterior and lateral teeth. The lower lateral teeth show a high and slender cusp, being broader at it base (Figs. 4W and 4X). In profile view, the cusp is slightly sigmoidal. The cutting edges are smooth. The root is moderately high with long and well separated lobes; forming an angle of about 106º (Fig. 3W). The upper anterior tooth displays a high, slender and slightly distally curved cusp (Figs. 4Y and 4Z). The root is high with well-separated lobes, the distal lobe is missing. Our teeth show the typical diagnostic character of the species *Mitsukurina lineata* (i.e thin vertical folds along the lingual face) (*Cappetta, 2012*). The material described here resemble the dental characters observed in those teeth reported from Costa Rica (*Laurito et al., 2014*).

Odontaspididae *Müller & Henle, 1839*

*Carcharias* *Rafinesque, 1810*

*Carcharias* sp.

(Figs. 5A and 5B)

A single tooth (Table S1) was identified as *Carcharias* sp. The upper posterior tooth shows a triangular, bent distally and lingually curved cusp (Figs. 5A and 5B). The cutting edges are smooth. The distal cusplet is short and blunt (i.e. probably eroded) whereas the mesial one is broken. The root is short and broad with a well-developed nutritive furrow that separates the lingual protuberance. Considering the morphological characters described here, the tooth described here can be unambiguously identified as belonging to the genus *Carcharias* (*Cappetta, 2012*). However, the diagnostic characters of this species are not clear to identify at specific level; thus, we prefer to identify it at generic level until more material is available.

*Odontaspis Agassiz, 1843*

*Odontaspis* sp.

(Fig. 5C-K)

Twenty teeth (Table S1) were identified as *Odontaspis* sp. The lower anterior teeth show a high and slender cusp with a sigmoidal profile (Fig. 5C-I). There are one to two pairs of lateral cusplets, which are very high and sharp. The cutting edges are smooth and do not reach the base of the cusp or the lateral cusplets. The root is high and robust with a lingual protuberance, which is divided by a nutritive groove. The lobes of the root are long and well-separated, forming an angle of about 85º. The upper posterior tooth displays a triangular and distally inclined cusp (Figs. 5J and 5K). Vertical folds are present at the base of the crown. A pair of lateral cusplets are preserved only in the distal section. The first lateral cusplet is very high, while the second one is smaller; both are very sharp. The root is short with both lobes missing; the lingual protuberance is less pronounced than in anterior files and divided by a nutritive groove. The teeth described here share the typical characters of the *Odontaspis* (*Cappetta, 2012*), however, we prefer to identify at generic level until more preserved material is available.

†Otodontidae *Glikman, 1964*

†*Otodus* *Agassiz, 1843*

†*Otodus* (C*archarocles*) cf. †*O. angustidens* (*Agassiz, 1843*)

(Fig. 5L-Q)

Five teeth (Table S1) were identified as *Otodus* (Carcharocles) cf. *O. angustidens.* The lower anterior tooth is incomplete; the preserved cutting edges is strongly serrated (Fig. 5L). The root is moderately high with a long mesial lobe whereas the distal one is broken. The upper anterior teeth show a triangular and straight cusp with strongly serrated cutting edges. (Fig. 5M-O). There are one pair of lateral cusplets, which are triangular and serrated. The root is moderately high with well-separated lobes. The upper lateral tooth displays a triangular and distally oriented cusp with strongly serrated cutting edges (Figs. 5P and 5Q). The preserved distal cusplet is triangular and also serrated. The root is incomplete but seems low with short lobes. The taxonomic classification of the megatoothed sharks has been the subject of debate for many years (*e.g. Cappetta, 2012*; *Pimiento & Balk, 2015*; *Perez et al., 2018*; *Boessenecker et al., 2019*). We followed *Cappetta* (*2012*) and recognize three subgenera within the genus *Otodus* to separate the different species of megatoothed sharks. For instance, in *Megaselachus*, the serrations are more regular and relatively smaller, whereas in *Otodus* the cutting edges are completely smooth. Additionally, differences in the root morphology are also used to separate those subgenera. The teeth described here resemble those teeth of *O. angustidens* reported from the late Oligocene of Europe and New Zealand (*Gottfried & Fordyce, 2001*).

†*Megalolamna* *Shimada et al., 2017*

†*Megalolamna paradoxodon Shimada et al., 2017*

(Fig. 5U-X)

Four teeth (Table S1) were identified as *Megalolamna paradoxodon.* The lower antero-lateral teeth show a high and straight cusp with smooth cutting edges (Figs. 5U and 5V). There are one pair of lateral cusplets, which are high and triangular. The root is high and robust with short and well-separated lobes. The lingual protuberance is moderately pronounced. The incomplete lateral tooth displays a short and distally inclined cusp with smooth cutting edges; the apex is missing (Fig. 5W-X). The preserved distal cusplet is robust, high and distally oriented, whereas the mesial one is broken. The root is moderately high with short and separated lobes. The teeth described here resemble those teeth of *M. paradoxodon* reported from the early Miocene of North America, Colombia and Peru (*Carrillo-Briceño et al., 2016b*; *Shimada et al., 2017*; *Landini et al., 2019*). According to *Landini et al* (*2019*), the upper tooth (described as Lamniformes gen. and sp. indet.) from Colombia can be unambiguously identified as *M. paradoxodon*.

Lamnidae *Müller & Henle, 1838*

Lamnidae indet.

(Fig. 6A-C)

One tooth (Table S1) was identified as Lamnidae indet*.* The lower anterior tooth shows a high and straight cusp with sigmoidal profile. The cutting edges are smooth and do not reach the base of the cusp. The labial and lingual faces of the crown are convex transversely; the enameloid surface is slightly ornamented by vertical folds at its medial and basal part on both faces. The lower part of the crown overhangs the lobes of the root by a slight bulge. The root is high and bulky with a strong lingual protuberance; the distal lobe is long, whereas the mesial one is broken. The tooth described here shows similarities with anterior teeth of *Isurolamna* from the late Oligocene of Europe (*Hovestadt, Hovestadt-Euler & Micklich, 2010*; *Cappetta, 2012*); however, more material is necessary to identify it at generic or specific levels. Therefore, we prefer to identify this tooth at family level until more material (e.g. lateral teeth) is available.

Carcharhiniformes *Compagno, 1973*

Carcharhinidae *Jordan & Evermann, 1896*

*Carcharhinus* *Blainville, 1816*

†*Carcharhinus gibbesii* *Woodward, 1889*

(Fig. 6N-S)

The sample is represented by 146 isolated teeth (Table S1). Upper teeth are the most abundant and these are characterized by low triangular crown slightly inclined distally in lateral and posterior teeth. Cutting edges are smooth, being the distal one slightly concave and the mesial slightly convex; a clear notch differentiate these from the heels. Both mesial and distal heels are rather straight and strongly serrated. The root is low with rounded and flattened lobes; in the lingual surface, a transverse narrow and well-defined groove reaches the base of the root. The lower teeth have a triangular crown marked by smooth cutting edges. There is no notch between the main cusp and the lateral heels, which bear completely smooth cutting edges. The root is low and its lobes rounded with a narrow medial lingual groove. The specimens from the Montañita-Olón site resemble those specimens reported from the late Oligocene of Europe (Reinecke et al., 2014), North America (*Cicimurri & Knight, 2009*) and early Miocene of Colombia (*Carrillo-Briceño et al., 2016b, 2019*) and Venezuela (*Carrillo-Briceño et al., 2016a*).

Batomorphii *Cappetta, 1980*

Myliobatiformes *Compagno, 1973*

Mobulidae *Gill, 1893*

*Mobula Rafinesque, 1810*

†*Mobula fragilis* (*Cappetta, 1970*)

(Fig. 8A-K)

Eight teeth (Table S1) were identified as *Mobula fragilis*. The presumed female antero-lateral teeth are mesio-distally elongated and labio-lingually compressed (Fig. 8A-K). The labial face of the crown is strongly ornamented by vertical ridges and grooves (Figs. 8A and 8E). The lingual face also show these ridges, but they do not reach the basal part of the crown (Fig. 8I). The occlusal surface is irregularly ornamented by vertical grooves (Figs. 8C and 8G). The crown has a roughly hexagonal contour and flat surface in occlusal view. The root is narrower and low; displaying the polyaulacorhizous vascularization type. According to *Adnet et al* (*2012*), the dental characters described here represent the group of mobulids with “cobblestone teeth”. Generally, the males have teeth with cuspidate crowns, whereas they are rounded in females. However, these dental characters are not always present or are less developed, depending on the species. The teeth described here are similar with those teeth of *M. fragilis* reported from late Oligocene of North America and early to middle Miocene of Venezuela (*Cicimurri & Knight, 2010*; *Carrillo-Briceño et al., 2016a*).

*Mobula* sp.

(Fig. 8L-R)

Two teeth (Table S1) correspond to *Mobula* sp. The teeth are square or rectangular shaped (Fig. 8L-R). In profile view, the crown is lingually oriented and slightly cuspidate with a rounded apex. The labial face of the crown is mostly smooth, being ornamented by vertical ridges on its upper region, whereas the lingual face is ornamented at its median part. The occlusal surface is ornamented by ridges and striations at its lower region. The root is broader and high; bilobed in specimen MPM-1384a (Fig. 8L) and trilobed in MPM-1384b (Fig. 8O). Small foramina are irregularly arranged on the lingual and labial faces of the root. The teeth described here can be unambiguously identified as belonging to the genus *Mobula* (*Adnet et al., 2012*; *Cappetta, 2012*; *Carrillo-Briceño et al. 2016a, Carrillo-Briceño et al. 2016b*). However, due the lack of diagnostic characters, we prefer to identify it at generic level until more material is available.

**References**

Agassiz L. 1833–1843. Recherches sur les poisons fossils. Neuchatel.

Applegate SP. 1972. A revision of the higher taxa of Orectoloboids. Journal of the Marine Biological Association of India 14(2): 743-751.

Barnard KH. 1925. A monograph of the marine fishes of South Africa. Part I (Amphioxus, Cyclostomata, Elasmobranchii, and Teleostei -Isospondyli to Heterosomata). Annals of the South African Museum 21: 1-418.

Berg LS. 1937. A classification of fish-like vertebrates. Bulletin of the Academy of Sciences of the USSR, Division of Chemical Science 1937: 1277-1280.

Blainville HMD. 1816. Prodrome d'une nouvelle distribution systematique du regne animal. Bulletin de la Société Philomathique de Paris 8: 05-112, 121-124.

Bleeker P. 1859. Enumeratio specierum piscium hucusque in Archipelago indico observatarum. Acta Societatis scientiarum Indo-Neerlandae 6: 1-276.

Bloch ME, Schneider JG. 1801. M.E. Blochii Systema Ichthyologiae iconibus ex illustratum. Post obitum auctoris opus inchoatum absolvit, correxit, interpolavit. Saxo: J.G. Schneide.

Boessenecker RW, Ehret DJ, Long DJ, Churchill M, Martin E, Boessenecker SJ. 2019. The Early Pliocene extinction of the mega-toothed shark Otodus megalodon: a view from the eastern North Pacific. PeerJ p. 7:e6088 DOI: https://doi.org/10.7717/peerj.6088.

Bonnaterre JP. 1788. Ichthyologie. Tableau encyclopédique et méthodique des trois règnes de la nature. Paris.

Buen FD. 1926. Catálogo ictiológico del Mediterráneo español y de Marruecos. Resultados Campañas Internaccionale Institute Español Oceanografíá 2: 153-161.

Cappetta H. 1970. Les Sélaciens du Miocène de la région de Montpellier. Palaeovertebrata, Mémoire Extraordinaire, no. 1970:1-139.

Cappetta H. 1980. Modification du satut generique de queleques especes de sélaciens crétacés et tertiares. Palaeovertebrata 10:29-42.

Cappetta H. 2012. Chondrichthyes. Mesozoic and Cenozoic Elasmobranchii: Teeth. Munich: Verlag Dr. Friedrich Pfeil.

Cappetta H, Gregorová R, Adnet S. 2016. New selachian assemblages from the Oligocene of Moravia (Czech Republic). Neues Jahrbuch für Geologie und Paläontologie-Abhandlungen 280(3):259-284 DOI:10.1127/njgpa/2016/0579.

Carrillo-Briceño JD, González-Barba G, Landaeta MF, Nielsen SN. 2013. Condrictios fósiles del Plioceno Superior de la Formación Horcón, Región de Valparaíso, Chile central. Revista Chilena de Historia Natural 86(2):191-206.

Carrillo-Briceño JD, De Gracia C, Pimiento C, Aguilera OA, Kindlimann R, Santamarina P, Jaramillo C. 2015. A new Late Miocene chondrichthyan assemblage from the Chagres Formation, Panama. Journal of South American Earth Sciences 60:56–70 DOI 10.1016/j.jsames.2015.02.001.

Carrillo-Briceño JD, Aguilera OA, De Gracia C, Aguirre-Fernández G, Kindlimann R, Sánchez-Villagra MR. 2016a. An early Neogene Elasmobranch fauna from the southern Caribbean (Western Venezuela). Palaeontologia Electronica 19.2.27A:1–32 DOI 10.26879/664.

Carrillo-Briceño JD, Argyriou T, Zapata V, Kindlimann R, Jaramillo CA. 2016b. A new Early Miocene (Aquitanian) Elasmobranchii assemblage from the Gaujira Peninsula, Colombia. Ameghiniana 53(2):77–99 DOI 10.5710/AMGH.26.10.2015.2931.

Carrillo-Briceño JD, Luz Z, Hendy A, Kocsis L, Aguilera O, Vennemann T. 2019. Neogene Caribbean elasmobranchs: diversity, paleoecology and paleoenvironmental significance of the Cocinetas Basin assemblage (Guajira Peninsula, Colombia). Biogeosciences 16(1):33-56 DOI: 10.5194/bg-16-33-2019.

Cicimurri DJ, Knight JL. 2009. Late Oligocene sharks and rays from the Chandler Bridge Formation, Dorchester County, South Carolina, USA. Acta Palaeontologica Polonica 54(4):627-647 DOI: doi: http://dx.doi.org/10.4202/app.2008.0077.

Compagno LJV. 1973. Interrelationships of living elasmobranchs. In: Greenwood, PH, Miles RS, Patterson C, ed. Interrelationships of fishes. London: Academic Press, 15-61.

Garman S. 1913. The Plagiostomia (Sharks, Skates and Rays). Memoirs of the Museum of Comparative Zoology at Harvard College 36: 1-528.

Gill T. 1862. Note on some genera of fishes of western North America. Proceedings of the Academy of Natural Sciences of Philadelphia 14:329-332.

Gill T. 1893. Families and subfamilies of fishes. Memoirs of the National Academy of Sciences: 125-138.

Glikman LS. 1964. Sharks of the Paleogene and their stratigraphic significance. Moscow: Nauka Press.

Goodrich ES. 1909. Vertebrata Craniata (First fascicle: Cyclostomes and Fishes). In Lankester R, ed. A treatise on Zoology. London: Adam and Charles Black, 1-518.

Gottfried MD, Fordyce RR. 2001. An associated specimen of *Carcharodon angustidens* (Chondrichthyes, Lamnidae) from the Late Oligocene of New Zealand, with comments on Carcharodon interrelationships. Journal of Vertebrate Paleontology 21(4):730-739 DOI: https://doi.org/10.1671/0272-4634(2001)021[0730:AASOCA]2.0.CO;2.

Gray J. 1851. List of the specimens of fish in the collection of the British Museum. Part 1. Chondropterygii. British Museum (Natural History).

Hovestadt DC, Hovestadt-Euler M, Micklich N. 2010. A review of the chondrichthyan fauna of Grube Unterfeld (Frauenweiler) clay pit. Kaupia 17:57-71.

Jordan DS 1898. Description of a species of fish (*Mitsukurina owstoni*) from Japan, the type of a distinct family of lamnoid sharks. Proceeding of the California Academy of Sciences 1:199-202.

Jordan DS, Evermann BW. 1896. The fishes of North and Middle America: a descriptive catalogue of the species of fish-like vertebrates found in the waters of North America, north of the Isthmus of Panama. Part I. Bulletin of the United States National Museum 47:1-1240.

Landini W, Collareta A, Di Celma C, Malinverno E, Urbina M, Bianucci G. 2019. The early Miocene elasmobranch assemblage from Zamaca (Chilcatay Formation, Peru). Journal of South American Earth Sciences 91:352-371 DOI: https://doi.org/10.1016/j.jsames.2018.08.004.

Laurito CA. 1999. Los seláceos fósiles de la localidad de Alto Guayacán (y otros ictiolitos asociados), Mioceno superior-Plioceno inferior de la Formación Uscari, provincia de Limón. Costa Rica, San José: Laurito, C. (Ed.).

Laurito MCA, Calvo C, Valerio Al, Calvo A, Chacón R. 2014. Ictiofauna del Mioceno inferior de la localidad de Pacuare de Tres Equis, Formación Río Banano, provincia de Cartago, Costa Rica, y descripción de un nuevo género y una nueva especie de scaridae. Revista Geológica de América Central 50:153-192.

Long DJ. 1993. Preliminary list of the marine fishes and other vertebrate remains from the Late Pleistocene Palos Verdes Sand Formation at Costa Mesa, Orange County, California. PaleoBios 15(1): 9-13.

Müller J, Henle J. 1837. Gattungen der Haifische und Rochen nach einer von ihm mit Hrn. Henle unternommenen gemeinschaftlichen Arbeit über die Naturgeschichte der Knorpelfische. Akademie der Wissenschaften zu Berlin 1837(2): 111-118.

Müller J, Henle J. 1838. Ueber die Gattungen der Plagiostomen. Archiv für Naturgeschichte 4:83-85.

Müller J, Henle J. 1839-1841. Systematische Beschreibung der Plagiostomen. Berlin.

Perez VJ, Godfrey SJ, Kent BW, Weems RE, Nance JR. 2018. The transition between Carcharocles chubutensis and Carcharocles megalodon (Otodontidae, Chondrichthyes): lateral cusplet loss through time. Journal of Vertebrate Paleontology 38(6): e1546732 DOI: 10.1080/02724634.2018.1546732.

Pfeil FH. 1983. Zahnmorphologische Untersuchungen an rezenten und fossilen Haien der Ordnungen Chlamydoselachiformes und Echinorhiniformes. Palaeo Ichthyologica 1:1-315.

Pimiento C, Balk MA. 2015. Body-size trends of the extinct giant shark Carcharocles megalodon: a deep-time perspective on marine apex predators. Paleobiology 41(3):479-490 DOI: 10.1017/pab.2015.16.

Probst J. 1879. Beiträge zur Kenntniss der fossilen Fische aus der Molasse von Baltringen. Hayfische. Jahreshefte des Vereins für vaterländische Naturkunde in Württemberg 35: 127-191.

Purdy R, Clellan JHM, Schneider VP, Applegate SP, Meyer R, Slaughter R. 2001. The Neogene sharks, rays and bony fishes from Lee Creek Mine, Aurora, North Carolina. Smithsonian Contributions to Paleobiology 90:71-202.

Rafinesque CS. 1810. Caratteri di alcuni nuovi generi e nouve specie di Animali e Piante della Sicilia con varie osservazioni sopra I medesimi. Palermo: Per le stampe di Sanfilippo.

Reed D. 1946. New species of fossil shark from New Jersey. Notulae Naturae of the Acadamy of Natural Sciences of Philadelphia 172:1-3.

Reinecke T, Balsberger M, Beaury B, Pollerspoeck J. 2014. The elasmobranch fauna of the Thalberg Beds, early Egerian (Chattian, Oligocene), in the Subalpine Molasse Basin near Siegsdorf, Bavaria, Germany. Palaeontos 26:1-127.

Shimada K, Chandler RE, Lam OLT, Tanaka T, Ward DJ. 2017. A new elusive otodontid shark (Lamniformes: Otodontidae) from the lower Miocene, and comments on the taxonomy of otodontid genera, including the ‘megatoothed’ clade. Historical Biology 29(5):704-714 DOI: 10.1080/08912963.2016.1236795.

Smith A. 1829. Contributions of the natural history of South Africa. Zoological Journal 4(54):433–444.

Staig F, Hernández S, López P, Villafaña JA, Varas C, Soto LP, Carrillo Briceño JD. 2015. Late Neogene elasmobranch fauna from the Coquimbo Formation, Chile. Revista Brasileira de Paleontologia 18:261-272 DOI: 10.4072/rbp.2015.2.07.

Vialle N, Adnet S, Cappetta H. 2011. A new shark and ray fauna from the Middle Miocene of Mazan, Vaucluse (southern France) and its importance in interpreting the paleoenvironment of marine deposits in the southern Rhodanian Basin. Swiss Journal of Palaeontology 130(2):241-258 DOI: 10.1007/s13358-011-0025-4.

Welton BJ. 1974. *Heptranchias Howellii* (Reed, 1946) (Selachii, Hexanchidae) in the Eocene of the United States and British Columbia. Paleobios 17:1-15.

Welton BJ. 1979. Late Cretaceous and Cenozoic Squalomorphii of the Northwest Pacific Ocean. PhD Thesis, University of California.

Woodward AS. 1889. Catalogue of the fossil fishes in the British Museum. Part. I. London: British Museum (Natural History).
