# Supplementary material for: Diversity and paleoenvironmental implications of an elasmobranch assemblage from the Oligocene–Miocene boundary of Ecuador
Source: PeerJ. 2020 Apr 29;8:e9051. doi: 10.7717/peerj.9051 (PMC7195833; doi:10.7717/peerj.9051)
Supplement: Supplemental Information 2 [file peerj-08-9051-s002.docx]

| **Fossil taxa** | **N° specimens** | **Extant species** | **Bathymetric range (m)** | | |
| --- | --- | --- | --- | --- | --- |
|  |  |  | **Min** | **Max** | **Common** |
| **Squalomorphii** |  |  |  |  |  |
| *Heptranchias* cf. †*H*. *howellii* | 28 | *Heptranchias perlo* | 0 | 1000 | 180-450 |
| *Hexanchus* cf. *H*. *griseus* | 31 | *Hexanchus* spp. | 1 | 2500 | 180-1100 |
| *Centrophorus* cf. *C*. *granulosus* | 46 | *Centrophorus* *granulosus* | 50 | 1440 | 200-600 |
| *Dalatias* sp. | 1 | *Dalatias licha* | 37 | 1800 | 200-1800 |
| cf. *Echinorhinus* sp. | 1 | *Echinorhinus* spp. | 10 | 1100 | 70-900 |
| †*Paraechinorhinus* cf. †*P*. *barnesi* | 1 | **―** |  |  |  |
| *Pristiophorus* sp. | 23 | *Pristiophorus* spp. | 1 | 1000 | 100-300 |
| **Galeomorphii** |  |  |  |  |  |
| *Rhincodon* sp. | 1 | *Rhincodon typus* | 0 | 1928 | 0-100 |
| *Isurus* cf. *I*. *oxyrinchus* | 7 | *Isurus oxyrinchus* | 0 | 750 | 100-150 |
| Lamnidae indet. | 1 | **―** |  |  |  |
| *Mitsukurina* cf. †*M*. *lineata* | 9 | *Mitsukurina owstoni* | 30 | 1300 | 270-960 |
| *Carcharias* sp. | 1 | *Carcharias taurus* | 1 | 191 | 15-25 |
| *Odontaspis* sp. | 20 | *Odontaspis ferox* | 10 | 2000 | 13-880 |
| †*Otodus* (*Carcharocles*) cf. †*O. angustidens* | 5 | **―** |  |  |  |
| †*Parotodus benedenii* | 2 | **―** |  |  |  |
| †*Megalolamna paradoxodon* | 3 | **―** |  |  |  |
| *Alopias* cf. †*A. exigua* | 5 | *Alopias* spp. | 0 | 730 | 0-200 |
| †*Alopias latidens* | 4 | *Alopias* spp. | 0 | 730 | 0-200 |
| †*Carcharhinus gibbesii* | 146 | *Carcharhinus* spp. | 0 | 1000 | < 80 |
| †*Galeocerdo aduncus* | 13 | *Galeocerdo cuvier* | 0 | 371 | < 150 |
| †*Physogaleus contortus* | 12 | **―** |  |  |  |
| †*Hemipristis serra* | 2 | *Hemipristis elongatus* | 0 | 130 | < 30 |
| *Sphyrna* sp. | 25 | *Sphyrna* spp. | 0 | 1000 | < 50 |
| indet. (articulated vertebrae) | 25 | **―** |  |  |  |
| **Batomorphii** |  |  |  |  |  |
| †*Mobula fragilis* | 8 | *Mobula*spp. | 0 | > 1000 | < 100 |
| *Mobula* sp. | 2 | *Mobula* spp. | 0 | > 1000 | < 100 |
| Indet. | 2 | **―** |  |  |  |

**R script for the analysis**

## analysis using no. specimens as weight and mean difference: mean.common - mean total

#load: boot

samplewmean<-function(d,i,j) {

d<-d[i,]

w<-j[i,]

return(weighted.mean(d,w))

}

#bat3 is a complete dataset

#bat4 is a subset that contains taxa name "taxa", specimens counts "No.specimens" and the difference between total range and common range means "difmeans"

bat4<-bat3[,c(1,2,9)]

names(bat4)

#[1] "taxa" "No.specimens" "difmeans"

#compute the absolute values to remove negative signs

bat4.1<-bat4[,2:3]

bat4.1[]<-lapply(bat4.1,abs)

bat4<-cbind(bat4[,1],bat4.1)

bat4

#weighted bootstraping where weight is given by the No. specimens in col 2

results2<-boot(data=bat4[,3,drop=FALSE],statistic = samplewmean,R=10000,j=bat4[,2,drop=FALSE])

#write the results

results2

#calculate the weighted mean to compare

weighted.mean(bat4[,3],bat4[,2])

#histogram plot

bottable<-results2$t

hist(bottable, breaks = 150, xlab = 'Water Depht',col = 'black', ylab = 'number of replicate samples',main = 'Ecuador sharks')

abline(v= 148.937,col="blue")

#confidence intervals

boot.ci(results2,conf = 0.95,type='Percentile')
