# Supplementary material for: Diversity and paleoenvironmental implications of an elasmobranch assemblage from the Oligocene–Miocene boundary of Ecuador
Source: PeerJ. 2020 Apr 29;8:e9051. doi: 10.7717/peerj.9051 (PMC7195833; doi:10.7717/peerj.9051)
Supplement: Supplemental Information 3 — Abbreviations: indeterminate (Indet.). [file peerj-08-9051-s003.docx]

| **Taxa** | **N° specimens** | **Elements** | **Jaw position** | | | **Teeth measurements (mm)** | | |
| --- | --- | --- | --- | --- | --- | --- | --- | --- |
|  |  |  | **Lower** | **Upper** | **Indet.** | **Height** | **Width** | **Length** |
| **Squalomorphii** |  |  |  |  |  |  |  |  |
| *Heptranchias* cf. †*H. howellii* | 28 | Teeth | 23 | 5 |  | 6,7-7 | 12,1-10 | **―** |
| *Hexanchus* cf. *H*. *griseus* | 31 | Teeth | 21 | 10 |  | 6,5-8,6 | 11,6-18,8 | **―** |
| *Centrophorus* cf. *C. granulosus* | 46 | Teeth | 42 | 4 |  | 2,3-5 | 2,7-4,6 | **―** |
| *Dalatias* sp. | 1 | Tooth | 1 |  |  | **―** | 4 | **―** |
| cf*. Echinorhinus* sp. | 1 | Tooth |  |  | 1 | **―** | **―** | **―** |
| †*Paraechinorhinus* cf. †*P*. *barnesi* | 1 | Tooth |  |  | 1 | 9 | 11 | **―** |
| *Pristiophorus* sp. | 23 | Teeth |  |  | 23 | **―** | **―** | 7-23,68 |
| **Galeomorphii** |  |  |  |  |  |  |  |  |
| *Rhincodon* sp. | 1 | Tooth |  |  | 1 | 6 | **―** | **―** |
| *Isurus* cf. *I*. *oxyrinchus* | 7 | Teeth |  |  | 7 | 12-24,5 | 15-15,24 | **―** |
| Lamnidae indet. | 1 | Tooth | 1 |  |  | 41 | 17 | **―** |
| *Mitsukurina* cf. †*M. lineata* | 9 | Teeth | 2 | 2 | 5 | 10,4-15,65 | 7,7-8,1 | **―** |
| *Carcharias* sp. | 1 | Tooth |  | 1 |  | 11,33 | 10,35 | **―** |
| *Odontaspis* sp. | 20 | Teeth |  |  | 20 | 9,5-28 | 3,5-12,5 | **―** |
| †*Otodus* (*Carcharocles*) cf. †*O. angustidens* | 5 | Teeth |  | 4 | 1 | 49,26 | 36,5-42,97 | **―** |
| †*Parotodus benedenii* | 2 | Teeth |  |  | 2 | 21 | 10.2 | **―** |
| †*Megalolamna paradoxodon* | 3 | Teeth |  |  | 3 | 16-25 | 17,53-21 | **―** |
| *Alopias* cf. †*A. exigua* | 5 | Teeth |  |  | 5 | 5,2-8 | 5,6-8 | **―** |
| †*Alopias latidens* | 4 | Teeth |  |  | 4 | 10,8-10, 9 | 12-12,81 | **―** |
| †*Carcharhinus gibbesii* | 146 | Teeth | 48 | 98 |  | 5-11,21 | 7-15,15 | **―** |
| †*Galeocerdo aduncus* | 13 | Teeth |  |  | 13 | 9,9-12 | 16,8-19,2 | **―** |
| †*Physogaleus contortus* | 12 | Teeth | 2 |  | 10 | 11,4-14,9 | 10,4-19,3 | **―** |
| †*Hemipristis serra* | 2 | Teeth |  | 2 |  | 18,77-21,50 | 22,8-23,51 | **―** |
| *Sphyrna* sp. | 25 | Teeth | 5 | 14 | 6 | 3,7-8,6 | 8,1-11,9 | **―** |
| Indet. | 25 | Vertebrae | **―** | **―** | **―** | **―** | **―** | **―** |
| **Batomorphii** |  |  |  |  |  |  |  |  |
| †*Moluba fragilis* | 8 | Teeth |  |  | 8 | 3,8-5,4 | 5,6-4,6 | 1,7-2,3 |
| *Moluba* sp. | 2 | Teeth |  |  | 2 | 1,5-2 | 2-2,5 | 1,2-1,5 |
| Indet. | 2 | Caudal spine | **―** | **―** | **―** | **―** | **―** | **―** |
