# Supplementary material for: Diversity and paleoenvironmental implications of an elasmobranch assemblage from the Oligocene–Miocene boundary of Ecuador
Source: PeerJ. 2020 Apr 29;8:e9051. doi: 10.7717/peerj.9051 (PMC7195833; doi:10.7717/peerj.9051)
Supplement: Supplemental Information 4 — Abbreviations: minimun (Mn), maximun (Mx), meters (m) and indeterminate (Indet.). Lifestyle: Benthic (B), Benthopelagic (Bp); Pelagic (P). Preferred Habitat Neritic (N), Oceanic (O), Sublitoral (S), Bathyal (Bt), Abysal (Ab), Hadal (Ha), Epipelagic (E), Mesopelagic (M), Bathypelagic (Bp). [file peerj-08-9051-s004.docx]

| **Taxa** | **N° specimens** | **Extant species** | **Lifestyle** | **Preferred habitat** | **Bathymetric range (m)** | | |
| --- | --- | --- | --- | --- | --- | --- | --- |
|  |  |  |  |  | **Min** | **Max** | **Common** |
| **Squalomorphii** |  |  |  |  |  |  |  |
| *Heptranchias* cf. †*H*. *howellii* | 28 | *Heptranchias perlo* | Bp | Bt/M | 0 | 1000 | 180-450 |
| *Hexanchus* cf. *H*. *griseus* | 31 | *Hexanchus* spp. | Bp | Bt/Bp | 1 | 2500 | 180-1100 |
| *Centrophorus* cf. *C*. *granulosus* | 46 | *Centrophorus* *granulosus* | Bp | Bt/Bp | 50 | 1440 | 200-600 |
| *Dalatias* sp. | 1 | *Dalatias licha* | Bp | Bt/Bp | 37 | 1800 | 200-1800 |
| cf. *Echinorhinus* sp. | 1 | *Echinorhinus* spp. | Bp | Bt/M | 10 | 1100 | 70-900 |
| †*Paraechinorhinus* cf. †*P*. *barnesi* | 1 | **―** |  |  |  |  |  |
| *Pristiophorus* sp. | 23 | *Pristiophorus* spp. | Bp | N/S/Bt | 1 | 1000 | 100-300 |
| **Galeomorphii** |  |  |  |  |  |  |  |
| *Rhincodon* sp. | 1 | *Rhincodon typus* | P | N/O/Bp | 0 | 1928 | 0-100 |
| *Isurus* cf. *I*. *oxyrinchus* | 7 | *Isurus oxyrinchus* | P | O/M | 0 | 750 | 100-150 |
| Lamnidae indet. | 1 | **―** |  |  |  |  |  |
| *Mitsukurina* cf. †*M*. *lineata* | 9 | *Mitsukurina owstoni* | Bp | Bt | 30 | 1300 | 270-960 |
| *Carcharias* sp. | 1 | *Carcharias taurus* | Bp | N/O | 1 | 191 | 15-25 |
| *Odontaspis* sp. | 20 | *Odontaspis ferox* | Bp | Bt/M | 10 | 2000 | 13-880 |
| †*Otodus* (*Carcharocles*) cf. †*O. angustidens* | 5 | **―** |  |  |  |  |  |
| †*Parotodus benedenii* | 2 | **―** |  |  |  |  |  |
| †*Megalolamna paradoxodon* | 3 | **―** |  |  |  |  |  |
| *Alopias* cf. †*A. exigua* | 5 | *Alopias* spp. | P | O/M | 0 | 730 | 0-200 |
| †*Alopias latidens* | 4 | *Alopias* spp. | P | O/M | 0 | 730 | 0-200 |
| †*Carcharhinus gibbesii* | 146 | *Carcharhinus* spp. | P/Bp | N/O | 0 | 1000 | < 80 |
| †*Galeocerdo aduncus* | 13 | *Galeocerdo cuvier* | Bp | N/O | 0 | 371 | < 150 |
| †*Physogaleus contortus* | 12 | **―** |  |  |  |  |  |
| †*Hemipristis serra* | 2 | *Hemipristis elongatus* | Bp | N | 0 | 130 | < 30 |
| *Sphyrna* sp. | 25 | *Sphyrna* spp. | P/Bp | N/O/M | 0 | 1000 | < 50 |
| indet. (articulated vertebrae) | 25 | **―** |  |  |  |  |  |
| **Batomorphii** |  |  |  |  |  |  |  |
| †*Mobula fragilis* | 8 | *Mobula* spp. | Bp | N/O/M | 0 | > 1000 | < 100 |
| *Mobula* sp. | 2 | *Mobula* spp. | Bp | N/O/M | 0 | > 1000 | < 100 |
| Indet. | 2 | **―** |  |  |  |  |  |
