# Supplementary material for: Diversity and paleoenvironmental implications of an elasmobranch assemblage from the Oligocene–Miocene boundary of Ecuador
Source: PeerJ. 2020 Apr 29;8:e9051. doi: 10.7717/peerj.9051 (PMC7195833; doi:10.7717/peerj.9051)
Supplement: Supplemental Information 5 [file peerj-08-9051-s005.docx]

| **Taxa** | **N° specimens** | **Elements** | **Catalog numbers** |
| --- | --- | --- | --- |
|  |  |  |  |
| **Squalomorphii** |  |  |  |
| *Heptranchias* cf. †*H. howellii* | 28 | Teeth | MPM-1365 (*MO-86) |
| *Hexanchus* cf. *H*. *griseus* | 31 | Teeth | MPM-1359 (*MO-27) |
| *Centrophorus* cf. *C. granulosus* | 46 | Teeth | MPM-1367 (*MO-88) |
| *Dalatias* sp. | 1 | Tooth | MPM-1366 (*MO-87) |
| cf. *Echinorhinus* sp. | 1 | Tooth | MPM-1368 (*MO-89) |
| †*Paraechinorhinus* cf. †*P*. *barnesi* | 1 | Tooth | MPM-1369 (*MO-90) |
| *Pristiophorus* sp. | 23 | Teeth | MPM-1361 (*MO-44) |
| **Galeomorphii** |  |  |  |
| *Rhincodon* sp. | 1 | Tooth | MPM-1370 (*MO-91) |
| *Isurus* cf. *I*. *oxyrinchus* | 7 | Teeth | MPM-1363 (*MO-79), MPM-1364 (*MO-80) |
| Lamnidae indet. | 1 | Tooth | MPM-1377 (*MO-98) |
| *Mitsukurina* cf. †*M*. *lineata* | 9 | Teeth | MPM-1371 (*MO-92) |
| *Carcharias* sp. | 1 | Tooth | MPM-1372 (*MO-93) |
| *Odontaspis* sp. | 20 | Teeth | MPM-1355 (*MO-09) |
| †*Otodus* (*Carcharocles*) cf. †*O. angustidens* | 5 | Teeth | MPM-1352 (*MO-06), MPM-1353 (*MO-07), MPM-1356 (*MO-15), MPM-1360 (*MO-34), MPM-1373 (*MO-94) |
| †*Parotodus benedenii* | 2 | Teeth | MPM-1357 (*MO-17) |
| †*Megalolamna paradoxodon* | 3 | Teeth | MPM-1350 (*MO-4), MPM-1351 (*MO-5), MPM-1378 (*MO-99) |
| *Alopias* cf. †*A. exigua* | 5 | Teeth | MPM-1374 (*MO-95) |
| †*Alopias latidens* | 4 | Teeth | MPM-1375 (*MO-96) |
| †*Carcharhinus gibbesii* | 146 | Teeth | MPM-1376 (*MO-97) |
| †*Galeocerdo aduncus* | 13 | Teeth | MPM-1379 (*MO-100) |
| †*Physogaleus contortus* | 12 | Teeth | MPM-1380 (*MO-101) |
| †*Hemipristis serra* | 2 | Teeth | MPM-1354 (*MO-08) |
| *Sphyrna* sp. | 25 | Teeth | MPM-1381 (*MO-102) |
| Indet. | 25 | Vertebrae | MPM-1382 (*MO-103) |
| **Batomorphii** |  |  |  |
| †*Moluba fragilis* | 8 | Teeth | MPM-1383 (*MO-104) |
| *Moluba* sp. | 2 | Teeth | MPM-1384 (*MO-105) |
| Indet. | 2 | Caudal spine | MPM-1385 (*MO-106) |
