# Supplementary figures and images for: Diversity and paleoenvironmental implications of an elasmobranch assemblage from the Oligocene–Miocene boundary of Ecuador
Source: PeerJ. 2020 Apr 29;8:e9051. doi: 10.7717/peerj.9051 (PMC7195833; doi:10.7717/peerj.9051)

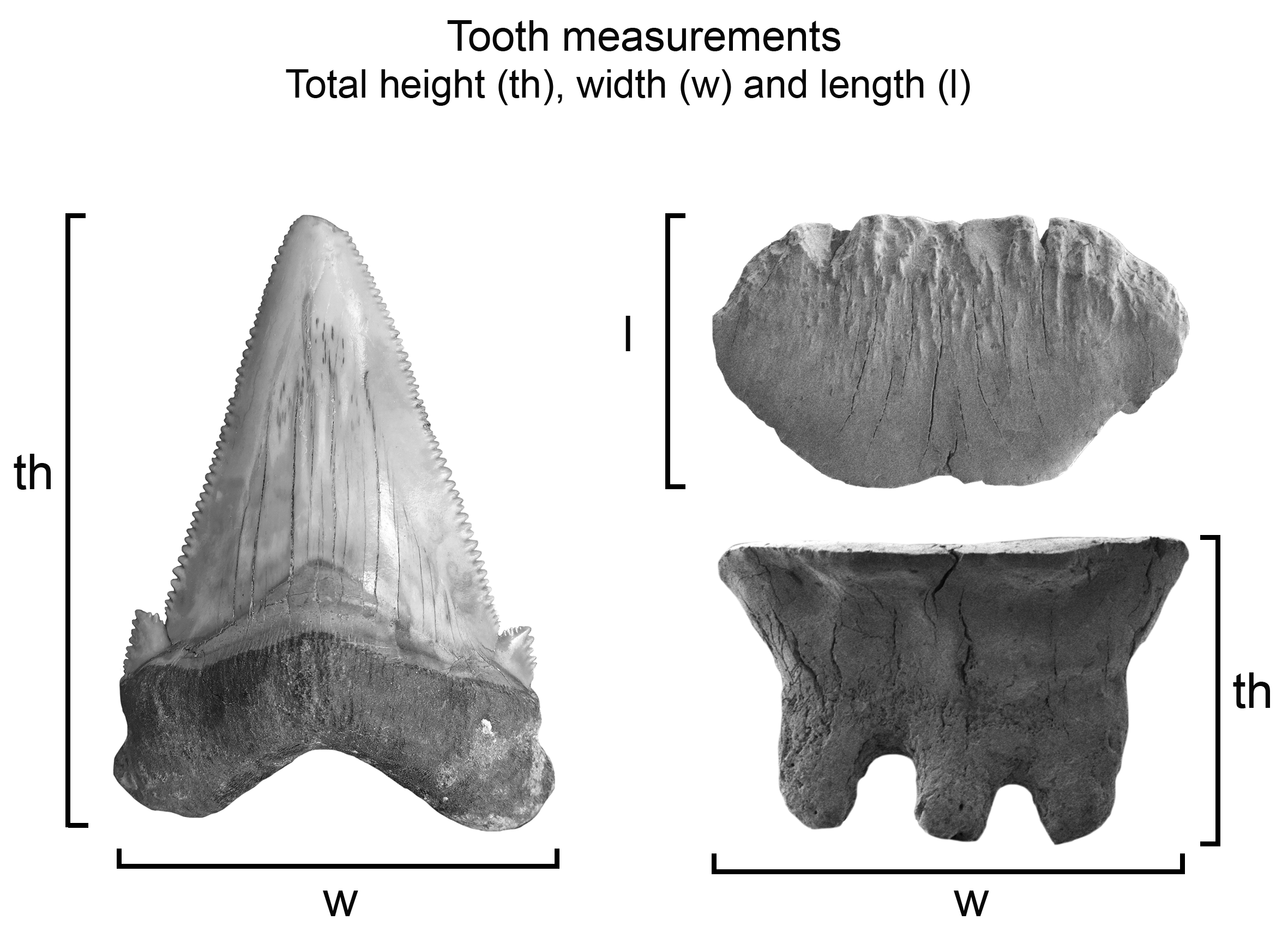

Supplement: Supplemental Information 6 [file peerj-08-9051-s006.png]

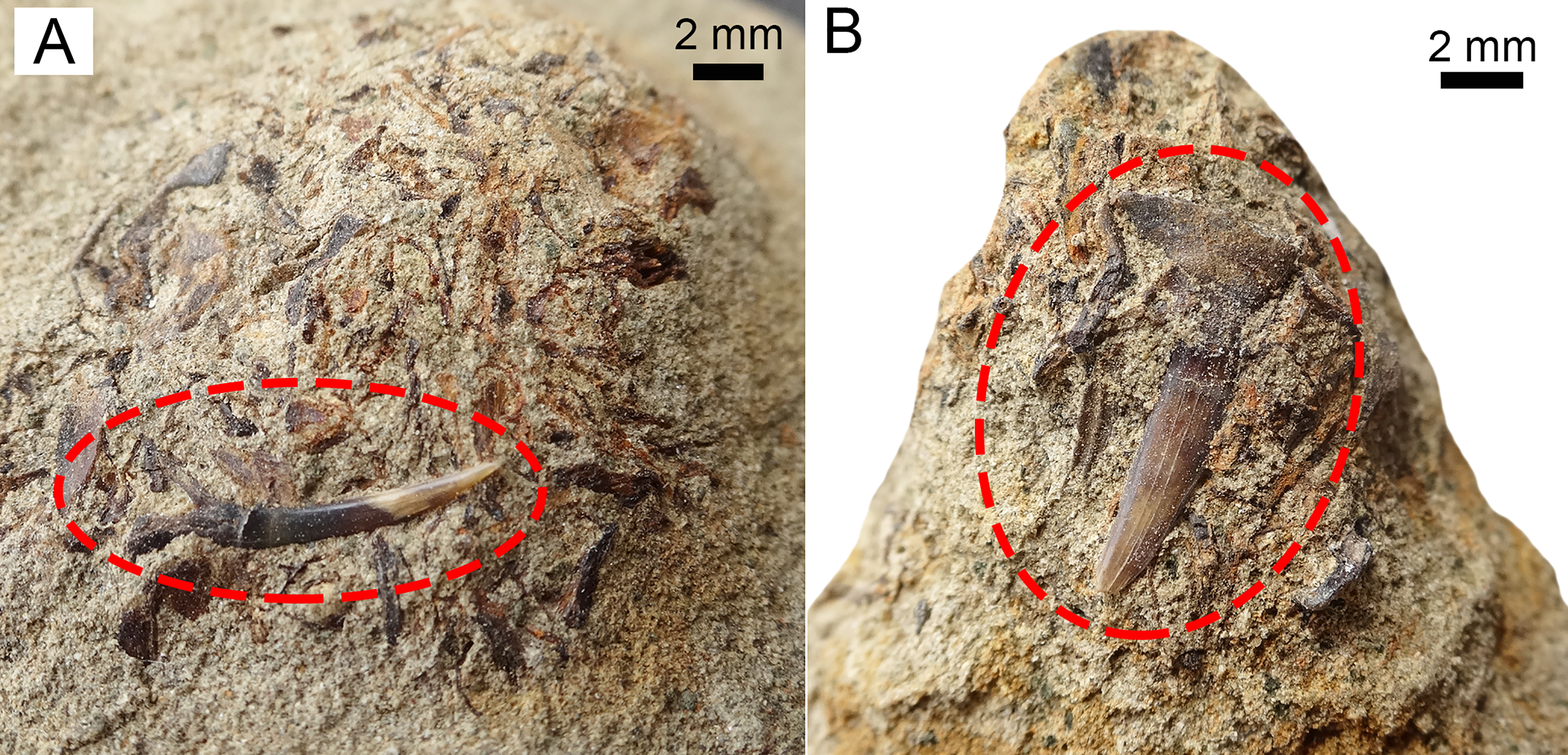

Supplement: Supplemental Information 7 — A. MPM-1389 and B. MPM-1390) with isolated rostral teeth of Pristiophorus sp. and indet. bony fish remains. [file peerj-08-9051-s007.png]
